# Photoreceptor Vulnerability to Ferroptosis: Membrane Phospholipid Peroxidation, Mitochondrial Homeostasis, and RPE–Photoreceptor Coupling

**DOI:** 10.3390/cimb48060616

**Published:** 2026-06-15

**Authors:** Yue Sun, Zhaorui Xu, Yanxia Wu, Mingxu Zhang, Xuejing Lu

**Affiliations:** 1 Eye School, Chengdu University of Traditional Chinese Medicine, Chengdu 610075, China; dr.sunmoon@stu.cdutcm.edu.cn (Y.S.); zrzr0619@stu.cdutcm.edu.cn (Z.X.); wuyanxia@cdutcm.edu.cn (Y.W.); 2 Eye Health with Traditional Chinese Medicine Key Laboratory of Sichuan Province, Chengdu 610075, China

**Keywords:** photoreceptors, ferroptosis, lipid peroxidation, mitochondrial homeostasis, retinal pigment epithelium, RPE–PR coupling, polyunsaturated fatty acids

## Abstract

Photoreceptor (PR) degeneration is a shared pathological feature of multiple blinding retinal diseases. This narrative review examines the mechanisms underlying PR vulnerability to ferroptosis-associated lipid-peroxidation injury, with emphasis on three interconnected features: the marked enrichment of docosahexaenoic acid (DHA) and other polyunsaturated fatty acids (PUFAs) in PR outer-segment disc membranes; the chronically high metabolic demand of PRs and the specialized spatial organization of their mitochondria; and retinal pigment epithelium (RPE)–PR metabolic coupling, including outer-segment renewal and phagocytic turnover, glucose transport and lactate shuttling, and visual-cycle–related all-trans-retinal (atRAL) clearance and bisretinoid accumulation. We also summarize antioxidant defense systems centered on the cystine/glutamate antiporter (xCT)–glutathione (GSH)–glutathione peroxidase 4 (GPX4) axis and mitochondrial GPX4 (mtGPX4), which restricts iron-dependent lipid peroxidation in PRs. We propose that highly oxidizable membrane phospholipid substrates, mitochondrial homeostatic imbalance, and impaired RPE–PR metabolic coupling may collectively shape PR susceptibility to ferroptosis-associated injury. From a therapeutic perspective, this framework supports multitarget strategies designed to interrupt lipid-peroxidation propagation, stabilize mitochondrial redox homeostasis and quality-control mechanisms, and restore RPE–PR metabolic support and local iron-buffering capacity.

## 1. Introduction

Progressive photoreceptor (PR) injury and loss constitute a major pathological basis for irreversible visual dysfunction in many blinding retinal diseases. Age-related macular degeneration (AMD) is one of the leading causes of blindness among older adults [1]. In 2021, approximately 8.06 million people worldwide were affected by AMD-related visual impairment, and this number is projected to rise to 21.34 million by 2050 [2]. In advanced AMD, outer-retinal atrophy or neovascular exudation is associated with photoreceptor degeneration and central vision loss, leading to marked impairment of quality of life [3]. Retinitis pigmentosa (RP) is a common inherited retinal degenerative disease with a global prevalence of approximately 1 in 5000 [4]. Despite its marked genetic heterogeneity [5], progressive PR degeneration remains a central pathological hallmark of RP [6]. Diabetic retinopathy (DR), a major ocular complication of diabetes, is projected to affect approximately 161 million people worldwide by 2045 [7].

Beyond classic microvascular abnormalities, accumulating evidence indicates that DR is also accompanied by early neuroretinal injury, in which PR dysfunction may precede overt microvascular lesions and further contribute to disease progression [8,9]. Importantly, DR remains clinically defined and staged mainly by vascular manifestations, whereas early diabetic retinal neurodegeneration may represent an additional and potentially independent target for intervention [10]. In this context, AMD therapy remains largely stage- and phenotype-dependent. For neovascular AMD, first-line intravitreal anti-vascular endothelial growth factor (VEGF) therapy includes ranibizumab, aflibercept, brolucizumab, and faricimab, with bevacizumab widely used off-label in clinical practice [11,12]. These agents mainly suppress VEGF-driven choroidal neovascularization, vascular permeability, and exudation, but do not directly target degenerative processes in early or dry AMD, including progressive photoreceptor dysfunction [13].

Similarly, broadly effective therapies that delay or halt PR degeneration in RP remain limited [14]. Therefore, elucidating the molecular basis of PR degeneration, particularly the upstream mechanisms that determine PR susceptibility to injury, is crucial for developing novel neuroprotective strategies. Since ferroptosis was first defined in 2012 [15], this iron-dependent, lipid-peroxidation-driven form of cell death has been increasingly implicated in the onset and progression of multiple retinal diseases, including AMD, RP, and DR [16]. Studies in PR-related models have shown that solute carrier family 7 member 11 (SLC7A11) downregulation weakens antioxidant defense and exacerbates ferroptosis-associated injury [17]. In diabetic models, iron burden and lipid peroxidation accompany PR injury; in inherited degeneration models, ferroptosis/oxytosis-related pathways have also been implicated [18,19]. Impaired all-trans-retinal (atRAL) clearance likewise induces Fe^2+^ accumulation, glutathione (GSH) depletion, and amplification of membrane lipid peroxidation [20]. Collectively, these findings suggest that ferroptosis may represent an important mechanism linking metabolic imbalance, oxidative stress, and PR degeneration. However, apoptosis remains the most extensively studied mode of PR death, and direct PR-specific evidence for ferroptosis, as well as clearer mechanistic stratification, is still needed [21]. In PR-like 661W cells and other retinal neuronal injury models, interventions that reduce ferroptosis-related changes have shown protective effects [22,23].

These observations raise the question of why PRs are particularly susceptible to lipid peroxidation under stress conditions. Several distinctive structural and metabolic features may help explain this susceptibility. First, PR outer-segment disc membranes are among the retinal membrane structures most highly enriched in docosahexaenoic acid (DHA) and other polyunsaturated fatty acids (PUFAs). This specialized lipid composition is essential for phototransduction, membrane fluidity, and disc renewal [24], but it also provides abundant substrates for lipid-peroxidation chain reactions [25,26]. Second, PRs are among the most metabolically demanding neurons in the retina, and cone-enriched regions, including the macula, show greater pyruvate metabolism and tricarboxylic acid cycle activity [27]. At the same time, the dense and highly ordered mitochondrial populations within the PR inner segment, particularly in the cone ellipsoid region, indicate that mitochondria not only support energy conversion but also help maintain photoreceptor structure and optical function [28,29].

Recent ultrastructural studies of human rod photoreceptors further reveal a specialized inner-segment architecture with distinctive mitochondrial positioning, suggesting that PR mitochondrial architecture is highly specialized and may vary across photoreceptor subtypes [30]. Although this sustained, high-flux, highly organized state supports visual signal transduction, it also suggests that, when mitochondrial dynamics, quality control, iron handling, or membrane homeostasis are disturbed, PRs may become particularly vulnerable to the accumulation of mitochondrial reactive oxygen species (mtROS), mitochondrial membrane-potential dysregulation, and lipid peroxidation. Third, PRs reside within an outer-retinal metabolic microenvironment shaped by the retinal pigment epithelium (RPE) [31]. The RPE mediates the phagocytosis, degradation, and partial lipid recycling of photoreceptor outer segments (POS) [32,33].

Through visual-cycle–related retinaldehyde handling and outer-retinal retinoid metabolism, the RPE also promotes atRAL clearance and helps limit related toxic stress, thereby maintaining local homeostasis [34]. When post-phagocytic POS processing is impaired, substrate allocation becomes imbalanced, or retinoid metabolism is disrupted, lipid-peroxidation-related and ferroptosis-promoting stress may accumulate within the RPE–PR metabolic unit and be further amplified [20]. Against this background, the cystine/glutamate antiporter (xCT)–GSH–glutathione peroxidase 4 (GPX4) axis constitutes the core molecular defense against iron-dependent lipid peroxidation [35,36,37]. Mitochondrial GPX4 (mtGPX4), in particular, is critical for limiting the accumulation of mitochondria-associated peroxidized phospholipids and for maintaining homeostasis in PUFA-enriched membrane systems [38,39].

Recent reviews have already established ferroptosis as a relevant mechanism in ocular and retinal diseases, covering topics ranging from eye-wide ferroptosis-related processes to disease-focused discussions of dry AMD, AMD-associated regulatory networks and therapeutic opportunities, and RP-associated photoreceptor injury [6,40,41,42]. Collectively, these studies provide an important foundation for understanding ferroptosis in ocular pathology. Building on this literature, there remains a need for a more focused synthesis of why photoreceptors (PRs) may represent a particularly vulnerable cellular compartment for ferroptosis-associated lipid-peroxidation injury. The present review adopts a more focused PR-centered perspective. Rather than attempting a comprehensive survey of ferroptosis across ocular tissues or within individual disease settings, it integrates findings from AMD, RP, DR, light-induced retinal degeneration, and other outer-retinal stress models to develop a PR-centered vulnerability framework. Within this framework, PR susceptibility to ferroptosis-associated lipid-peroxidation injury is interpreted not merely as the consequence of a single cell-death pathway, but as a vulnerability state shaped by the convergence of three interrelated features: enrichment of highly oxidizable membrane lipid substrates, high mitochondrial bioenergetic demand and dependence on mitochondrial homeostatic integrity, and reliance on RPE–PR metabolic coupling.

Accordingly, this review first outlines the membrane-lipid, mitochondrial, and RPE–PR coupling mechanisms that may contribute to PR susceptibility to ferroptosis-associated injury. It then summarizes core defense pathways, including the xCT–GSH–GPX4 axis and mitochondrial GPX4, and discusses how disturbances in mitochondrial dynamics, mitochondrial quality control, biogenesis, and iron homeostasis may further increase PR vulnerability. Finally, the review evaluates potential intervention strategies aimed at interrupting lipid-peroxidation propagation, stabilizing mitochondrial redox and quality-control mechanisms, and restoring RPE–PR metabolic support and local iron-buffering capacity.

### Literature Search and Selection Strategy

This article is a narrative review with a focused mechanistic scope. To improve the transparency of literature selection, we performed structured searches of PubMed/MEDLINE and Web of Science Core Collection, supplemented by manual screening of reference lists from relevant original articles and reviews. Searches for ferroptosis-related evidence primarily covered peer-reviewed English-language publications from January 2012, when ferroptosis was first defined, to May 2026, with a final manual update in June 2026 to include newly available studies highly relevant to the review scope. Earlier foundational studies were included when they provided essential mechanistic context for photoreceptor membrane phospholipid composition, PUFA-containing phospholipid homeostasis, mitochondrial homeostasis, RPE–photoreceptor metabolism, visual-cycle biology, or retinal degeneration, but these studies were not treated as direct ferroptosis evidence.

Search terms were constructed around four domains and combined using the Boolean operators “AND” and “OR”: (1) ferroptosis-related terms, including “ferroptosis”, “lipid peroxidation”, “phospholipid peroxidation”, “phospholipid hydroperoxide”, “GPX4”, “SLC7A11”, “xCT”, “ACSL4”, “iron overload”, “ferritinophagy”, and “ferroptosis inhibitor”; (2) photoreceptor/outer-retina terms, including “photoreceptor”, “rod”, “cone”, “outer retina”, “outer segment”, “retinal pigment epithelium”, and “RPE”; (3) susceptibility-related mechanisms, including “DHA”, “PUFA”, “PUFA-containing phospholipid”, “mitochondria”, “mitochondrial ROS”, “mitochondrial dynamics”, “mitophagy”, “mitochondrial iron”, “visual cycle”, “retinaldehyde”, “glucose”, and “lactate”; and (4) disease/model terms, including “age-related macular degeneration”, “retinitis pigmentosa”, “diabetic retinopathy”, “light-induced retinal degeneration”, “Stargardt disease”, “Abca4”, “Rdh8”, “661W”, “ARPE-19”, “sodium iodate”, “NaIO_3_”, “all-trans-retinal”, “atRAL”, “A2E”, “RSL3”, “erastin”, and “tert-butyl hydroperoxide”. For therapeutic and translational sections, additional terms were used when relevant, including “ferrostatin-1”, “Fer-1”, “MitoTEMPO”, “deferiprone”, “deferoxamine”, “N-acetylcysteine”, “NAC”, “lutein”, “zeaxanthin”, “TSPO”, “BCL-2”, “BH3 mimetic”, “mitochondrial permeability transition pore”, “mPTP”, “cyclosporin A”, “elamipretide”, “voretigene neparvovec”, “ciliary neurotrophic factor”, “CNTF”, and “revakinagene taroretcel”.

Eligible studies included peer-reviewed original research articles, translationally relevant studies, animal and cellular models, and review articles that addressed ferroptosis-associated lipid peroxidation or mechanistically related redox, iron, mitochondrial, lipid-metabolic, or RPE–photoreceptor processes. Priority was given to photoreceptor-specific, retinal, or outer-retinal evidence. Non-retinal studies were selectively included only when they provided mechanistic insight relevant to photoreceptor ferroptosis vulnerability, such as mitochondrial dynamics, mitochondrial iron handling, mitochondrial membrane phospholipid homeostasis, or ferroptosis-defense systems; such evidence is discussed as indirect or hypothesis-generating where appropriate. Background epidemiological, clinical, therapeutic, guideline-based, and regulatory sources were also included when necessary to contextualize disease burden, current treatment limitations, approved indications, and translational relevance, but they were not used as direct mechanistic evidence for photoreceptor ferroptosis. Studies were excluded if they were outside the mechanistic scope of the review, did not address ferroptosis-associated lipid peroxidation or relevant redox/metabolic mechanisms, lacked full-length evidence, represented duplicate publications, or were available only as conference abstracts, editorials, or commentaries without original mechanistic or clinically relevant information. Because this article is a narrative review rather than a systematic review or meta-analysis, no PRISMA flow diagram, quantitative evidence synthesis, or formal risk-of-bias assessment was performed.

## 2. Biological Basis of Photoreceptor Susceptibility to Ferroptosis

### 2.1. Lipid Basis

PR outer-segment disc membranes have a highly specialized lipid composition; they are among the most highly polyunsaturated biological membrane structures in the retina and are particularly enriched in DHA [24]. This lipid specialization is essential for maintaining the architecture of outer-segment disc membranes and PR homeostasis. Mechanistically, the lipid composition of PR outer-segment membranes is established through the coordinated regulation of fatty acid supply, selective esterification, and phospholipid remodeling. Acyl-CoA synthetase long-chain family member 6 (ACSL6) supports DHA enrichment and retention in retinal membrane phospholipids and is critical for shaping the phospholipid composition of rod photoreceptors; *Acsl6* inactivation markedly reduces DHA-containing retinal phospholipids and leads to progressive rod photoreceptor loss [43].

Multifunctional protein 2 (MFP2) deficiency causes DHA insufficiency and early degenerative changes in both PRs and the RPE, supporting a role for peroxisomal β-oxidation in maintaining retinal DHA availability and outer-retinal integrity [44]. Likewise, loss of lysophosphatidic acid acyltransferase 3 (LPAAT3) reduces DHA levels in retinal membrane phospholipids, disrupts PR outer-segment disc organization, and impairs visual function [45]. Together, these findings indicate that DHA is required not only for shaping membrane composition but also for maintaining disc membrane ultrastructure. Accordingly, lipid homeostasis in PR outer-segment membranes depends on the coordinated action of multiple pathways that govern lipid metabolism and membrane organization.

Notably, the burden of oxidizable membrane lipid substrates also differs among PR subtypes. Compared with cone-dominant samples, rod-dominant samples contain higher levels of PUFAs, including long-chain and very-long-chain polyunsaturated fatty acids (LC- and VLC-PUFAs), suggesting intrinsic subtype-specific differences in the substrates available for peroxidation [25]. At the molecular level, DHA-related lipid–rhodopsin interactions can modulate rhodopsin conformational states and function through ligand-like and solvent-like mechanisms [46]. Although this membrane specialization supports visual function, it also renders outer-segment disc membranes chemically more vulnerable to oxidative attack. The outer retina is continuously exposed to light and resides in an oxygen-rich environment, where PUFAs are particularly susceptible to reactive oxygen species (ROS)-mediated lipid peroxidation chain reactions because of their multiple double bonds [26]. Collectively, these features create a highly PUFA-enriched, substrate-rich membrane environment that is permissive for lipid peroxidation in PR outer segments.

### 2.2. Mitochondrial Basis

PRs are major contributors to the high energy demand of the outer retina and require substantial adenosine triphosphate (ATP) to support dark-current-associated ion transport, phototransduction-related homeostasis, and synaptic transmission [47,48]. Despite their pronounced aerobic glycolytic phenotype, PRs remain highly dependent on mitochondrial metabolism. Metabolomic studies show that, compared with rod-enriched regions, cone-enriched retinal regions, particularly the macula, exhibit greater pyruvate metabolism, tricarboxylic acid cycle activity, and lipid synthesis [27], indicating higher demands for mitochondrial oxidative metabolism and biosynthetic support in cone-dominant retinal regions. Consistent with this, loss of glutamic-oxaloacetic transaminase 1 (GOT1) disrupts cytosolic–mitochondrial metabolic coupling and leads to PR degeneration [49], underscoring the importance of intact mitochondrial metabolism for PR homeostasis.

Beyond their high metabolic demands, PR mitochondria also exhibit a spatial organization closely aligned with cellular function. Recent studies of human rod photoreceptors have identified an accessory inner segment that lies adjacent to the outer segment and extends along its lateral aspect, with mitochondria positioned at the base of this accessory structure [30]. In cone photoreceptors, mitochondria are densely concentrated within the ellipsoid region of the inner segment, where they form highly ordered bundles immediately adjacent to the base of the outer segment; this organization has been proposed to facilitate local light focusing and guide incident light, thereby enhancing photon delivery to the outer segments [28]. Three-dimensional reconstructions of primate photoreceptors further demonstrate that inner-segment mitochondria exhibit a dense and highly ordered spatial arrangement, a configuration consistent with the high bioenergetic demands and specialized functional architecture of PRs [29].

Moreover, optic atrophy 1 (OPA1) has been linked to the orderly spatial alignment of neighboring PR mitochondria [50], suggesting that the maintenance of this specialized organization depends, at least in part, on mitochondrial dynamics. Taken together, PR mitochondria function under sustained high metabolic flux within a highly ordered spatial architecture. These features underscore the dependence of PRs on intact mitochondrial homeostasis. When mitochondrial homeostasis is disrupted, PRs may become more vulnerable to redox imbalance and mitochondrial dysfunction, thereby increasing their susceptibility to lipid peroxidation- and ferroptosis-associated injury.

### 2.3. RPE–PR Metabolic Coupling

PRs reside at the outer-retinal interface, where the RPE, Bruch’s membrane, and the choroid form a tightly coupled structural and metabolic unit. Within this interface, the apical–basal polarity of the RPE determines nutrient influx, metabolite efflux, and outer-segment turnover, thereby providing the structural basis for metabolic homeostasis in the outer retina [31]. Under physiological conditions, new discs are generated at the base of the outer segment, whereas aged discs are shed from the distal tip and phagocytosed by the RPE. The RPE mediates the recognition, phagocytosis, and lysosomal degradation of POS and contributes to partial lipid recycling [32,33]. Outer-segment phagocytosis and processing are also closely linked to circadian regulation in the RPE [51].

Following POS uptake, post-phagocytic processing in the RPE couples outer-segment clearance with POS-derived lipid handling, including fatty-acid oxidation and ketogenesis, while β-hydroxybutyrate release after outer-segment phagocytosis further suggests that POS turnover contributes to RPE metabolic adaptation [52]. Defective RPE mitochondrial energetics and impaired oxidative metabolic programs may further disrupt RPE metabolic homeostasis in the context of outer-retinal disease [53]. Moreover, RPE phagocytosis can promote local insulin production and influence retinal glucose uptake and homeostasis, further indicating that POS turnover actively participates in the outer-retinal metabolic ecosystem [54]. Thus, RPE–PR coupling involves both disc clearance and post-phagocytic substrate handling in the RPE, thereby contributing to a continuous system of lipid turnover and substrate reallocation in the outer retina [55].

**Figure 1:**
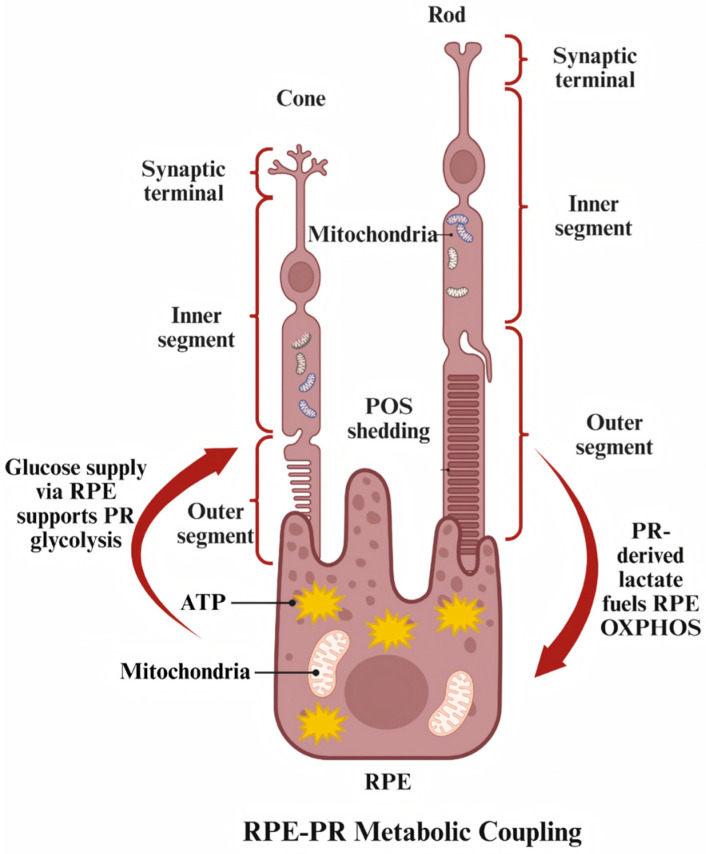
Structural and metabolic coupling between photoreceptors and the retinal pigment epithelium in the outer retina. Abbreviations: ATP, adenosine triphosphate; OXPHOS, oxidative phosphorylation; POS, photoreceptor outer segment; PR, photoreceptor; RPE, retinal pigment epithelium. Created in BioRender. sun, Y. (2026). https://BioRender.com/uubk9n6, accessed on 7 June 2026.

Recent in vivo isotope-tracing evidence provides direct flux-based support for this metabolic exchange, indicating that glucose is a major retinal fuel, that PRs are a major glucose-consuming population, and that PR-derived lactate can be transported to and catabolized by the RPE [56]. These data support the directionality and relative metabolic routing of glucose-to-PR and lactate-to-RPE exchange, rather than defining a universal percentage or fixed absolute flux of PR-derived lactate oxidation across species, developmental stages, or disease states. Earlier biochemical evidence and review-level synthesis further suggest that the RPE–retina metabolic ecosystem is fuel-flexible and context-dependent [57,58]. Together, these observations support viewing glucose transfer and lactate handling as variable components of RPE–PR metabolic coupling that help maintain outer-retinal substrate allocation and redox buffering. The structural and metabolic organization of the RPE–PR unit is summarized in Figure 1. Within this metabolic framework, glucose is not merely an energy source for PRs. It also provides carbon skeletons for outer-segment renewal and related biosynthetic processes, and restricted glucose uptake leads to defective outer-segment renewal and shortening of rod outer segments [59,60].

In addition, glucose utilization in PRs depends on the maintenance of specific metabolic programs. Glycolysis sustained by pyruvate kinase M2 (PKM2) and hexokinase 2 (HK2) supports central carbon flux and helps maintain PR structural integrity, function, and long-term survival [61,62,63,64]. More broadly, recent reviews emphasize that metabolic regulation is central to PR survival, development, and repair [65]. At the transport level, the monocarboxylate transporter 1 (MCT1) and monocarboxylate transporter 3 (MCT3), together with their shared chaperone basigin (BSG), mediate lactate transport across the RPE–PR interface; disruption of this pathway perturbs outer-retinal metabolic homeostasis and compromises PR function [66]. Beyond nutrient exchange, the RPE also supports visual-cycle–related retinoid handling at the PR–RPE interface. Retinal pigment epithelium-specific 65 kDa protein (RPE65) participates in the regeneration of 11-*cis*-retinoids and is therefore critical for visual pigment renewal and sustained photosensitivity [34]. Collectively, the RPE and PRs are metabolically coupled through continuous outer-segment lipid turnover, energy-substrate exchange, and visual-cycle–related retinoid handling. Together, these processes sustain outer-retinal homeostasis.

## 3. Antioxidant and Lipid-Redox Defense Systems Against Ferroptosis

### 3.1. xCT–GSH–GPX4 Axis-Mediated Antioxidant Defense in Photoreceptor Ferroptosis

The cystine/glutamate antiporter system xc^−^, composed of the light-chain transporter subunit SLC7A11 and the heavy-chain subunit solute carrier family 3 member 2 (SLC3A2), mediates cystine uptake in exchange for glutamate export and thereby supports cysteine availability for GSH biosynthesis [35]. After entering the cell, cystine is reduced to cysteine and incorporated into GSH, which supplies reducing equivalents for GPX4-dependent lipid peroxide detoxification. GPX4 directly reduces phospholipid hydroperoxides in biological membranes to their corresponding lipid alcohols, thereby limiting the propagation of lipid-peroxidation chain reactions and suppressing ferroptosis [67,68]. Thus, either reduced cystine supply or impaired GPX4 activity weakens the cellular defense against iron-dependent lipid peroxidation and increases susceptibility to ferroptosis. xCT deficiency alters local cysteine/GSH homeostasis in the retina and is associated with redox imbalance and altered mitochondrial activity, indicating that this transport system plays an important role in maintaining local antioxidant homeostasis in retinal tissue [69].

In PR-like 661W cell models, sodium iodate (NaIO_3_) treatment is associated with decreased SLC7A11 expression, GSH depletion, elevated Fe^2+^ levels, ROS accumulation, and increased lipid peroxidation; exogenous GSH, ferrostatin-1 (Fer-1), and N-acetylcysteine (NAC) alleviate NaIO_3_-induced cell death and ferroptosis-associated phenotypes, suggesting that disruption of xCT–GSH-dependent antioxidant defense contributes to ferroptosis-associated injury in PR-like cells [70]. In high-glucose-treated 661W PR-like cells and diabetic mouse retinas, downregulation of SLC7A11 and GPX4 is accompanied by an increased iron burden and elevated ROS and malondialdehyde levels, whereas Fer-1 partially reverses these changes [18]. These findings suggest that impairment of the xCT–GSH–GPX4 axis may contribute to diabetic retinal injury and may be relevant to PR vulnerability. Findings obtained from 661W cells should be interpreted with caution because this immortalized cone photoreceptor-like cell line, while retaining selected photoreceptor-related features, has also been reported to exhibit retinal ganglion precursor-like properties and does not fully reproduce mature primary PRs or the in vivo retinal microenvironment. Accordingly, 661W-based evidence is treated in this review as supportive in vitro evidence rather than direct evidence from primary PRs or in vivo models [71,72]. More recent studies further indicate that SLC7A11 downregulation weakens antioxidant defense and aggravates ferroptosis-associated injury in PR-related models, reinforcing the view that SLC7A11 is a critical determinant of the integrity of the xCT–GSH–GPX4 axis [17]. In the rd10 model of inherited PR degeneration, ferroptosis-related genes are upregulated, and ferroptosis inhibitors partially improve visual function, supporting a role for ferroptosis-related pathways in inherited PR degeneration [19].

Beyond the canonical xCT–GSH–GPX4 axis, upstream regulators may also shape PR susceptibility to ferroptosis by affecting the stability of this pathway. Studies have shown that cullin 7 (CUL7) downregulation reduces ubiquitin-mediated GPX4 degradation and attenuates ferroptosis-associated PR injury [73]. In light-damage models, lipocalin-2 (LCN2) inhibition is associated with reduced Fe^2+^ and MDA levels, restored SLC7A11 and GPX4 expression, and attenuated ferroptosis-associated injury in PRs [74]. Overall, current evidence supports the xCT–GSH–GPX4 axis as a core molecular defense against iron-dependent lipid peroxidation in PRs. When this axis is disrupted, PR vulnerability to ferroptosis-associated injury may increase substantially.

### 3.2. mtGPX4-Mediated Mitochondrial Lipid-Redox Defense in Photoreceptors

GPX4 is essential for preserving the integrity of PR membrane systems and subcellular architecture. Ueta et al. reported that GPX4 was abundantly expressed in PR inner segments and colocalized with mitochondrial markers; in mice with PR-specific deletion of *Gpx4*, PRs still differentiated into rods and cones but rapidly developed outer-segment disorganization, shortened connecting cilia, and mitochondrial structural abnormalities [38]. Azuma et al. further showed that mtGPX4 deficiency caused marked retinal accumulation of peroxidation products derived from DHA-containing phosphatidylethanolamine (PE), leading to cone loss before maturation and gradual rod degeneration after maturation, ultimately producing a cone–rod dystrophy-like phenotype [39]. Together, these findings indicate that mtGPX4 provides more than general antioxidant protection. It is also critical for limiting the accumulation of mitochondria-associated peroxidized phospholipids and for maintaining redox homeostasis in membrane systems enriched in PUFAs.

To date, direct evidence for upstream regulation of mtGPX4 in PRs remains limited. Studies in PC12 and HT22 neuronal models provide indirect, hypothesis-generating evidence that sirtuin 3 (SIRT3) activation may help protect against neuronal ferroptosis by reducing mtGPX4 acetylation [75]. In the retina, Ban et al. reported that SIRT3 deficiency was associated with mitochondrial abnormalities in PR inner segments and aggravated PR injury after light-induced stress [76]. However, these findings do not establish a SIRT3–mtGPX4–ferroptosis pathway in PRs in vivo; therefore, the SIRT3–mtGPX4 connection should currently be viewed as a potential upstream regulatory hypothesis requiring PR-specific validation. By contrast, the available PR-focused evidence indicates that mtGPX4 is a mitochondrial antioxidant enzyme that directly limits mitochondria-associated phospholipid peroxides, thereby linking mitochondrial redox homeostasis to the integrity of PR membrane structures. Accordingly, when mtGPX4 function is impaired, mitochondrial phospholipid peroxides accumulate, thereby increasing PR susceptibility to iron-dependent lipid-peroxidation-driven injury.

### 3.3. Putative CoQ- and BH_4_-Dependent Ferroptosis Defense Pathways in Photoreceptors

In addition to the canonical xCT–GSH–GPX4 axis and mtGPX4, the dihydroorotate dehydrogenase (DHODH)–reduced coenzyme Q (CoQH_2_), ferroptosis suppressor protein 1 (FSP1)–coenzyme Q_10_ (CoQ_10_), and GTP cyclohydrolase 1 (GCH1)–tetrahydrobiopterin (BH_4_) systems have also been proposed as parallel or supplementary antiferroptotic defense pathways. However, direct evidence for these pathways in the retina, particularly in PRs, remains limited. DHODH is localized to the inner mitochondrial membrane and uses coenzyme Q (CoQ) as an electron acceptor during de novo pyrimidine synthesis, thereby generating the reduced form, CoQH_2_ [77,78]. The DHODH–CoQH_2_ axis is increasingly viewed as a mitochondrial lipid-redox defense that helps restrain mitochondrial lipid peroxidation and ferroptosis, acting alongside mtGPX4 and other CoQH_2_-generating mechanisms within a complementary defense network [79,80]. In studies of ocular tissues, however, direct evidence for a ferroptosis-suppressive role of DHODH remains scarce, and current data come mainly from hypoxia-stressed human corneal epithelial cell models [81]. Therefore, whether the DHODH–CoQH_2_ system functions as an effective mitochondrial antiferroptotic defense in PRs remains unresolved.

FSP1 uses nicotinamide adenine dinucleotide phosphate (NADPH) to reduce CoQ_10_ to ubiquinol, its reduced antioxidant form, thereby establishing a relatively GPX4-independent lipid radical-scavenging system [82,83]. In blue-light-induced retinal injury, imbalance of the GSH–GPX4 and FSP1–CoQ_10_–NADH defense systems was associated with ferroptosis-related retinal damage, whereas interventions targeting iron overload or ferroptosis attenuated lipid-peroxidation-related injury and retinal degeneration [84]. This link is further supported by models of choroidal neovascularization, in which vitamin K protected ferroptosis-stressed RPE cells through vitamin K epoxide reductase (VKOR)/FSP1-dependent mechanisms [85]. In photoreceptor-relevant retinal organoids, *AIFM2*, which encodes FSP1, was upregulated in mature retinol dehydrogenase 12 (RDH12)-associated organoids showing shortened photoreceptor segments, reduced photoreceptor abundance, and cone-related defects [86]. However, this organoid finding indicates disease-associated transcriptional remodeling in a photoreceptor-relevant context, but does not demonstrate that FSP1 protects native PRs from ferroptosis. Thus, the subcellular localization and protective function of FSP1 in native PRs still require dedicated genetic or pharmacological validation.

**Figure 2:**
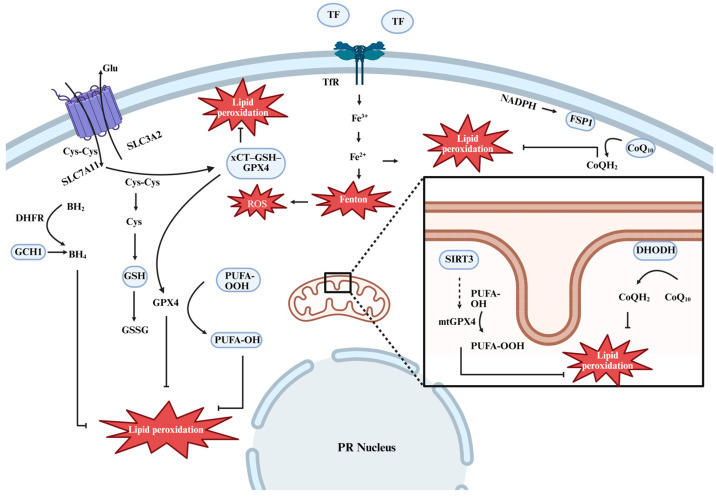
Core and putative ferroptosis-regulatory pathways relevant to photoreceptors. The schematic summarizes major pathways involved in ferroptosis-associated lipid peroxidation in PRs. The main panel shows cytosolic and plasma-membrane-associated modules, including xCT/SLC7A11-mediated cystine uptake, GSH-dependent GPX4 activity, TfR-associated iron input, Fenton chemistry, ROS generation, and the putative or supplementary FSP1–CoQ_10_/CoQH_2_ and GCH1–BH_4_/DHFR systems. The inset highlights mitochondrial lipid-redox regulation involving mtGPX4 and DHODH–CoQH_2_. Red starbursts indicate pro-ferroptotic oxidative events, blue ovals indicate enzymes, transporters, or metabolites with protective or regulatory functions, solid arrows indicate transport, conversion, or regulatory steps shown in the schematic, blunt-ended lines indicate inhibitory or detoxifying effects, and dashed arrows indicate indirect or hypothesis-generating links. Accordingly, the SIRT3–mtGPX4 relationship is presented as a putative upstream regulatory link rather than as a confirmed PR-specific ferroptosis mechanism, and the FSP1–CoQ_10_/CoQH_2_, DHODH–CoQH_2_, and GCH1–BH_4_/DHFR pathways should be interpreted as supplementary or putative antiferroptotic defenses whose direct PR-specific roles remain incompletely validated. Abbreviations: BH_2_, dihydrobiopterin; BH_4_, tetrahydrobiopterin; CoQ_10_, coenzyme Q_10_; CoQH_2_, reduced coenzyme Q_10_/ubiquinol; Cys, cysteine; Cys–Cys, cystine; DHFR, dihydrofolate reductase; DHODH, dihydroorotate dehydrogenase; Fe^2+^, ferrous iron; Fe^3+^, ferric iron; FSP1, ferroptosis suppressor protein 1; GCH1, GTP cyclohydrolase 1; Glu, glutamate; GPX4, glutathione peroxidase 4; GSH, reduced glutathione; GSSG, oxidized glutathione; mtGPX4, mitochondrial glutathione peroxidase 4; NADPH, reduced nicotinamide adenine dinucleotide phosphate; PR, photoreceptor; PUFA-OH, reduced polyunsaturated fatty acid-containing phospholipid alcohol; PUFA-OOH, polyunsaturated fatty acid-containing phospholipid hydroperoxide; ROS, reactive oxygen species; SIRT3, sirtuin 3; SLC3A2, solute carrier family 3 member 2; SLC7A11/xCT, solute carrier family 7 member 11/cystine–glutamate antiporter; TF, transferrin; TfR, transferrin receptor. Created in BioRender. sun, Y. (2026). https://BioRender.com/bikf196, accessed on 7 June 2026.

The GCH1–BH_4_ system is thought to suppress ferroptosis through a mechanism that is relatively independent of the GSH–GPX4 axis by maintaining BH_4_ levels and promoting lipid remodeling, thereby selectively preventing the depletion of peroxidation-prone PUFA-containing phospholipids [87]. In models of ischemic retinopathy, BH_4_ precursor supplementation improves redox status and reduces apoptotic cell death mainly in the inner neural retina, but this does not yet establish a direct PR-specific antiferroptotic role for the GCH1–BH_4_ system [88]. These core and putative antiferroptotic pathways are summarized schematically in Figure 2.

## 4. Mitochondrial Homeostasis as a Determinant of PR Susceptibility to Ferroptosis

### 4.1. Imbalance in Mitochondrial Dynamics

#### 4.1.1. Impaired Mitochondrial Fusion

Available evidence indicates that mitochondrial fusion contributes to the establishment of the specialized mitochondrial architecture and associated metabolic phenotype of rod photoreceptors [89]. Among the regulators involved in mitochondrial fusion, OPA1 is a key determinant of structural homeostasis in PR mitochondria. OPA1 mediates inner mitochondrial membrane fusion and cristae remodeling [90], and its loss in PR models leads to mitochondrial enlargement and disrupts the orderly alignment of adjacent mitochondria within the inner segment [50]. These findings indicate that OPA1 is closely linked to both the spatial organization of PR mitochondria and the structural stability of the inner mitochondrial membrane. Accordingly, OPA1 dysfunction may induce abnormal mitochondrial morphology and inner membrane disorganization, thereby potentially weakening the ability of PRs to adapt to metabolic and oxidative stress and increasing PR vulnerability to lipid-peroxidation-associated injury.

In addition to OPA1, mitofusin 2 (MFN2)-dependent maintenance of mitochondria–endoplasmic reticulum contact sites (MERCs) may represent another convergence point between impaired mitochondrial fusion and oxidative damage. Studies have shown that MFN2 dysfunction disrupts the structure and function of MERCs and impairs mitochondrial Ca^2+^ uptake through the inositol 1,4,5-trisphosphate receptor (IP3R) type 3 (IP3R3)–glucose-regulated protein 75 (GRP75)–voltage-dependent anion channel 1 (VDAC1) complex [91]. Evidence from other cell systems further suggests that MERC abnormalities can disrupt Ca^2+^ transport, alter ROS levels, and disturb ferroptosis-related lipid homeostasis [92]. In PR-related studies, IP3R type 2 (IP3R2)-mediated MERC-associated Ca^2+^ transport contributes to hypoxia-induced PR injury and is accompanied by mitochondrial dysfunction and increased cell death [93].

Related studies in SH-SY5Y neuroblastoma cells show that GPX4 inhibition promotes IP3R-mediated Ca^2+^ release, elevates mitochondrial Ca^2+^ levels, and drives ferroptosis [94]. Together, these findings suggest that impaired mitochondrial fusion may compromise PR resistance to oxidative stress by altering mitochondrial morphology and inner membrane structure, as well as by disrupting MERC-associated Ca^2+^ homeostasis and redox balance.

#### 4.1.2. Aberrant Mitochondrial Fission

Enhanced dynamin-related protein 1 (DRP1)-dependent mitochondrial fission may increase PR susceptibility to lipid-peroxidation-associated injury. In NIH-3T3 and HT-1080 cells, human NSCLC H441/A549 cells, *Drp1^fl/fl^* MEFs, and immortalized mouse hippocampal HT22 neurons, DRP1 activation or loss-of-function studies link ferroptosis induction to mitochondrial fragmentation, loss of membrane potential, and aggravated oxidative damage; conversely, DRP1 deficiency or deletion can partially delay these changes and preserve mitochondrial redox homeostasis [95,96]. Mechanistically, recent evidence indicates that mitochondrial fission is induced during ferroptosis and that disruption of mitochondrial dynamics, including interference with DRP1-dependent fission, can attenuate ferroptotic cell death, supporting a functional link between mitochondrial fission and ferroptosis-associated mitochondrial injury [97].

In retinal models, retinas from models of type 1 diabetes mellitus without overt diabetic retinopathy (T1DM-NDR), as well as high-glucose-treated 661W cells, exhibit increased DRP1 expression and phosphorylation, together with mitochondrial fragmentation, cristae disruption, reduced mitochondrial network branching, and loss of mitochondrial membrane potential [98]. Collectively, these findings suggest that excessive DRP1 activation impairs mitochondrial ultrastructural and functional homeostasis in retinal and PR-related models, aggravates oxidative injury, and thereby may increase susceptibility to ferroptosis-associated damage; however, conclusions based on 661W cells remain indirect and require PR-specific validation. Evidence from outer-retinal cells further supports this view. Studies of RPE cells derived from donors with AMD reveal disease-specific differences in mitochondrial fission and mitophagy responses, together with delayed recovery after stress, suggesting impaired restoration of mitochondrial homeostasis in outer-retinal cells [99].

Notably, damage associated with aberrant mitochondrial fission in PRs may extend beyond organelle-level energy imbalance and oxidative injury to include potential inflammatory amplification, although its connection with PR ferroptosis remains indirect. In non-retinal cancer-cell models, mitochondria-localized cyclic GMP–AMP synthase (cGAS) has been shown to promote DRP1 oligomerization and suppress ferroptosis-related mitochondrial ROS accumulation [100]. However, this cGAS–DRP1–ferroptosis mechanism has not been directly demonstrated in PRs. Retinal studies have shown that oxidative stress can activate cGAS–stimulator of interferon genes (STING) signaling in degenerating retinas, accompanied by cytosolic leakage of damaged DNA in PRs, and that inhibition of this pathway attenuates retinal inflammation and PR degeneration [101]. In retinal degeneration models, cGAS-dependent inflammatory activation has also been closely linked to DNA-induced microglial activation and PR degeneration [102]. Thus, cGAS–STING can be regarded as a putative inflammatory amplification pathway that may interact with mitochondrial stress, rather than as a directly established PR ferroptosis mechanism.

### 4.2. Insufficient Mitochondrial Quality Control

The PTEN-induced kinase 1 (PINK1)–Parkin pathway is a canonical mechanism of mitochondrial quality control. Loss of mitochondrial membrane potential activates PINK1/Parkin-dependent ubiquitin signaling on the outer mitochondrial membrane, thereby marking damaged mitochondria for autophagy–lysosomal clearance through the recruitment of autophagy adaptors [103,104]. Retinal studies suggest that selective mitochondrial clearance is an integral component of retinal development and remodeling. Two peaks of mitophagy have been observed during retinal development; the second is PINK1/Parkin-dependent and follows an increase in oxidative stress [105]. This finding indicates that the retina possesses an active mitochondrial quality-control program. By facilitating the recognition and clearance of damaged mitochondria while limiting their abnormal persistence, mitophagy serves as an important physiological mechanism for maintaining the homeostasis of the mitochondrial pool.

Consistent with this, PR-related cellular models further suggest that oxidative stress activates PINK1/Parkin-associated mitochondrial quality control. In H_2_O_2_-treated 661W cells, mitophagy is accompanied by increased colocalization of PINK1 with Parkin and of microtubule-associated protein 1 light chain 3 (LC3) with mitochondria [106]. In peroxisome proliferator-activated receptor gamma coactivator 1-alpha (PGC-1α)/nuclear factor erythroid 2-related factor 2 (NRF2; encoded by *NFE2L2*)-deficient models, RPE cells show elevated light chain 3 beta (LC3B), PINK1, and Parkin levels without a corresponding increase in autophagy–lysosome-mediated clearance [107]. This finding suggests that mitochondrial quality control in RPE cells may fail when the initiation of mitophagy is not matched by effective autolysosomal clearance; this state may be further aggravated by impaired mitochondrial biogenesis in *PGC-1α*/*NFE2L2*-deficient settings.

Beyond the canonical PINK1/Parkin pathway, the transient receptor potential mucolipin 1 (TRPML1)–transcription factor EB (TFEB) axis may provide an important auxiliary mechanism for mitochondrial quality control. TRPML1 activation promotes TFEB-dependent lysosomal biogenesis and mitophagy, thereby alleviating mitochondrial injury and improving redox homeostasis [108]. Moreover, beyond its role in LC3 recruitment, optineurin (OPTN) can promote phagosome maturation, facilitate phagosome–lysosome fusion, and enhance lysosome biogenesis by activating TRPML1-dependent TFEB, thereby helping RPE cells cope with the phagocytic burden imposed by outer segments [109]. These observations indicate that mitochondrial quality control in outer-retinal cells is closely linked to lysosomal function. Together, these findings indicate that mitochondrial quality control represents an important regulatory mechanism through which PRs and RPE cells respond to mitochondrial injury. When this quality-control network is inadequate, damaged mitochondria may persist abnormally, increasing outer-retinal susceptibility to oxidative stress and, plausibly, to ferroptosis-associated lipid peroxidation.

### 4.3. Insufficient Mitochondrial Biogenesis Capacity

In addition to mitochondrial dynamics and quality control, PRs must continuously replenish their mitochondrial pool to maintain mitochondrial homeostasis. PGC-1α is a canonical transcriptional coactivator of mitochondrial biogenesis that regulates the expression of nuclear-encoded mitochondrial proteins and transcriptional programs involved in oxidative metabolism and mitochondrial renewal [110]. In rd1 mice, the PGC-1α activator ZLN005 enhanced mitochondrial biogenesis, improved visual function, and delayed PR degeneration [111]. Evidence from a P23H retinitis pigmentosa model similarly showed that pharmacological upregulation of PGC-1α and mitochondrial transcription factor A (TFAM) was associated with activation of mitochondrial biogenesis and preservation of visual function, supporting the broader relevance of mitochondrial biogenesis in inherited PR degeneration [112].

At the level of mitochondrial gene expression, nuclear respiratory factor 1 (NRF1) and TFAM cooperate to support mitochondrial biogenesis and maintain mitochondrial DNA (mtDNA) expression. In a rod-specific *Nrf1*-knockout model, inner-segment mitochondria displayed abnormal morphology, aberrant subcellular positioning, and impaired function; these changes were accompanied by loss of TFAM expression and reduced cytochrome c oxidase (COX) activity and ultimately led to progressive rod degeneration and secondary cone loss [113]. These findings indicate that sustained mitochondrial renewal is essential for preserving mitochondrial function and PR survival.

In addition, impaired mitochondrial biogenesis in the RPE may further destabilize outer-retinal homeostasis. Inhibition of PGC-1α disrupts lipid metabolism and promotes lipid droplet accumulation in RPE cells [114]. In ARPE-19 cells, copper exposure increased peroxisome proliferator-activated receptor gamma coactivator 1-beta (PGC-1β) and TFAM expression, enhanced cytochrome c oxidase activity, and increased mitochondrial mass and mtDNA copy number, indicating that RPE respiratory-chain function is closely linked to mitochondrial biogenesis programs [115]. Because the RPE and PRs remain tightly coupled metabolically, insufficient mitochondrial renewal in the RPE may indirectly increase PR vulnerability to oxidative and metabolic stress by weakening RPE metabolic support and redox buffering capacity.

### 4.4. Mitochondrial Iron Burden and Disruption of Membrane Homeostasis

Mitochondria serve as key sites for iron–sulfur (Fe–S) cluster biogenesis and cellular iron regulation. Consequently, disturbances of iron import, utilization, or export can disrupt mitochondrial iron homeostasis and exacerbate oxidative stress [116,117]. In this context, transferrin receptor 1 (TfR1)-mediated iron uptake, ferritin heavy chain 1 (FTH1)- and ferritin light chain (FTL)-dependent ferritin storage, and NCOA4-mediated ferritinophagy collectively regulate the balance between ferritin-sequestered iron and the redox-active labile iron pool. In the retina, TfR1 and ferritin are detected not only in the RPE but also in photoreceptor inner segments or photoreceptor-enriched layers, and iron-dyshomeostasis models show altered TfR1 and H-/L-ferritin expression alongside photoreceptor dysfunction or degeneration [118]. By directing ferritin to the autophagy–lysosome pathway, NCOA4-mediated ferritinophagy can release ferritin-derived iron into the labile pool and may increase ferroptosis susceptibility under lipid-redox stress [119,120]. With respect to mitochondrial iron import, evidence from KU812 and K562 leukemia-cell models has identified the poly(rC)-binding protein 2 (PCBP2)–translocase of outer mitochondrial membrane 20 (TOM20)–sideroflexin 3 (SFXN3) axis as a potential route for cytosolic iron entry into mitochondria. Functionally, knockdown of PCBP2 or SFXN3 in these leukemia-cell systems reduces catalytic ferrous iron within mitochondria and mitochondrial respiratory capacity, whereas SFXN3 overexpression causes mitochondrial Fe^2+^ accumulation and increases sensitivity to RAS-selective lethal 3 (RSL3)-induced ferroptosis [121].

In the retina, *Sfxn3* mutations cause progressive outer retinal degeneration, including RPE thinning, suggesting that SFXN3 is required for outer-retinal homeostasis; whether this phenotype reflects altered mitochondrial iron handling or ferroptosis remains unresolved [122]. Mitochondrial membrane homeostasis may likewise influence PR susceptibility to iron-dependent lipid peroxidation. In the inner mitochondrial membrane, cardiolipin (CL) is a signature phospholipid that supports the organization and stability of respiratory-chain supercomplexes [123]. In CL-lacking yeast respiratory-supercomplex and PCBP1-depleted mouse-liver models, depletion of cardiolipin and CoQ is associated with impaired respiratory-chain function, reduced oxygen consumption, and insufficient ATP production [124]. These findings suggest that disruption of inner mitochondrial membrane lipid homeostasis may promote oxidative injury by impairing respiratory-chain function and weakening mitochondrial bioenergetic stability. Within this membrane-centered framework, retinal ferroptosis-associated injury can be identified experimentally by pairing oxidative or iron-dependent insults with a ferroptotic profile, including Fe^2+^ accumulation, GSH depletion, lipid peroxidation, and rescue by ferroptosis inhibitors or iron chelators. In ARPE-19 cells, tert-butyl hydroperoxide (t-BuOOH/tBHP) induces RPE ferroptosis-associated injury that is alleviated by Fer-1 or deferoxamine but exacerbated by exogenous iron loading [125]; in NaIO_3_-injured PR-relevant 661W cells, the injury phenotype similarly involves Fe^2+^ accumulation, GSH depletion, lipid-redox imbalance, and sensitivity to Fer-1-mediated protection [70]. Mitochondrial swelling assays using isolated mitochondria further support the link between iron-dependent lipid peroxidation and mitochondrial membrane injury: Fer-1 delays t-BuOOH/Fe^2+^-induced mitochondrial swelling, whereas butylated hydroxytoluene, bromoenol lactone and cyclosporin A more broadly preserve mitochondrial function by limiting lipid-radical propagation, calcium-independent phospholipase A2γ (iPLA2γ)-associated phospholipid hydrolysis, or cyclophilin D-dependent mPTP opening [126].

At the outer mitochondrial membrane, abnormal protein homeostasis and increased membrane permeability may further drive cells toward irreversible injury. In dibutyl phthalate-exposed zebrafish ZF4 cells, upregulation and oligomerization of voltage-dependent anion channel 2 (VDAC2) are accompanied by mitochondrial iron overload and loss of mitochondrial membrane potential, contributing to ferroptosis [127]. In BALB/c mice and neonatal rat cardiomyocytes exposed to trastuzumab-related cardiotoxic stress, the lipid peroxidation product 4-hydroxynonenal (4-HNE) can bind to VDAC1 and promote its oligomerization, leading to the opening of the mitochondrial permeability transition pore (mPTP), dissipation of membrane potential, and ATP depletion [128]. Consistently, in DU145 and 22Rv1 prostate cancer cells, VDAC1 oligomers can form aberrant channels in the outer mitochondrial membrane, increasing mitochondrial ROS, depleting GSH, dissipating membrane potential, and enhancing susceptibility to ferroptosis [129]. During cysteine deprivation or RSL3 exposure, VDAC1 oligomerization has been linked to mitochondrial dysfunction, mitochondrial ROS accumulation, and lipid peroxidation; in cysteine-deprived cells, pharmacological inhibition of VDAC1 oligomerization by NSC15364 or DIDS attenuates mitochondrial redox changes, reduces lipid peroxidation, and suppresses ferroptotic injury [130]. Although direct evidence in PRs remains limited, findings from non-PR systems, including trastuzumab-induced cardiomyopathy models, zebrafish ZF4 cells, prostate cancer cells, and cysteine deprivation- or RSL3-induced ferroptosis models, suggest that outer mitochondrial membrane channel remodeling can couple altered mitochondrial permeability to redox imbalance and regulated cell death. In retinal models, inhibition of VDAC1 oligomerization likewise alleviates mitochondrial dysfunction and attenuates PANoptosis-associated injury [131]. However, whether VDAC oligomerization directly contributes to PR ferroptosis remains unproven.

The 18 kDa translocator protein (TSPO) is an outer mitochondrial membrane protein increasingly implicated in retinal oxidative responses. In retinal degeneration, TSPO is enriched in reactive microglial populations and can modulate microglial inflammation and phagocytosis [132]. Mechanistically, photoreceptor debris can activate a TSPO–NADPH oxidase 1 (NOX1) axis in retinal phagocytes, driving Ca^2+^-dependent ROS production; treatment with the synthetic TSPO ligand XBD173 or microglia-specific TSPO deletion suppresses this neurotoxic oxidative response [133]. In PR-relevant models, topical PIGA1138, a TSPO ligand belonging to the N, N-dialkyl-2-arylindol-3-ylglyoxylamide (PIGA) class, preserved visual function, outer-retinal structure, and cone survival in rd10 mice, supporting PIGA1138 as a potential retinal protector [134]. Mechanistic support for this protective profile comes from lipopolysaccharide (LPS)-stressed 661W photoreceptor-like cells, where PIGA-class TSPO ligands attenuated inflammatory and apoptotic responses and reduced LPS-induced cytotoxicity; notably, SU-10603, a pregnenolone synthesis inhibitor, partially reversed PIGA-mediated cytoprotection, suggesting a role for TSPO-linked pregnenolone/neurosteroid production in this protective effect [135]. This neurosteroid-linked component is relevant to mitochondrial homeostasis because TSPO-ligand–promoted pregnenolone/neurosteroid production has been associated with improved mitochondrial respiration, ATP production, mitochondrial membrane-potential maintenance, and ROS control in neuronal models, thereby potentially increasing the tolerance of PRs and retinal glia to inflammatory or oxidative stress [136]. Together, the TSPO–PIGA–neurosteroid axis may act upstream of ferroptotic execution, functioning as a mitochondrial and inflammatory stress-buffering mechanism that could lower PR susceptibility to lipid-peroxidation-associated injury.

**Figure 3:**
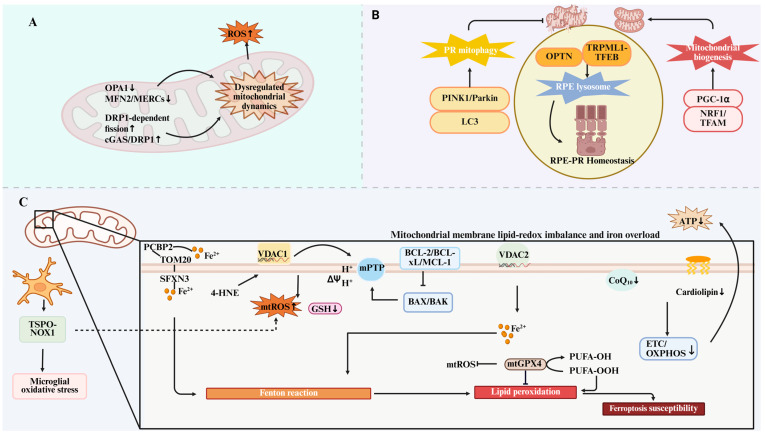
Mitochondrial homeostatic mechanisms that modulate photoreceptor susceptibility to ferroptosis-associated injury. The schematic is organized into three conceptual modules: (**A**) dysregulated mitochondrial dynamics in PRs, including OPA1-, MFN2/MERCs-, DRP1-, and cGAS/DRP1-related events; (**B**) the mitophagy–lysosome and mitochondrial-biogenesis axis functionally linked to the retinal pigment epithelium (RPE)–PR homeostatic unit, including PINK1/Parkin–LC3-mediated PR mitophagy, OPTN- and TRPML1–TFEB-related lysosomal regulation, and PGC-1α–NRF1/TFAM-dependent mitochondrial biogenesis; and (**C**) a magnified mitochondrial membrane-centered module showing mitochondrial iron handling, TSPO–NOX1-associated microglial oxidative-stress input, mtROS production, disturbance of mitochondrial membrane potential, BCL-2-family/BAX–BAK-related regulation of mitochondrial tolerance, mPTP opening/activation, depletion of GSH, CoQ_10_, and cardiolipin, impaired ETC/OXPHOS and ATP production, and mtGPX4-dependent reduction of PUFA-OOH to PUFA-OH. In panels (**A**,**C**), red/orange starbursts and red bars or boxes indicate oxidative stress, Fenton chemistry, lipid peroxidation, or ferroptosis-promoting outcomes, whereas the starburst labels in panel B denote conceptual homeostatic modules. Yellow/orange rounded boxes indicate regulatory or quality-control components; blue elements indicate lysosomal, pore-related, respiratory-function, or BCL-2-family-related mitochondrial-tolerance modules; pink/red-outlined elements indicate mitochondrial-biogenesis-related components; and green/gray elements indicate mitochondrial membrane, transport, or lipid-redox factors. Solid arrows indicate proposed directional relationships or downstream consequences, curved arrows indicate regulatory, trafficking, or feedback-related links, blunt-ended lines indicate inhibitory or detoxifying effects, upward or downward arrows indicate increased or reduced abundance, activity, or function, respectively, gray dashed lines separate conceptual modules rather than physical cellular barriers, and black dashed lines indicate indirect or hypothesis-generating links. The lower boxed region represents a magnified view of mitochondrial membrane-associated lipid-redox and iron-overload processes connected to the small mitochondrion on the left by black guide lines. Abbreviations: PR, photoreceptor; RPE, retinal pigment epithelium; ROS, reactive oxygen species; mtROS, mitochondrial reactive oxygen species; OPA1, optic atrophy 1; MFN2, mitofusin 2; MERCs, mitochondria–endoplasmic reticulum contact sites; DRP1, dynamin-related protein 1; cGAS, cyclic GMP–AMP synthase; PINK1, PTEN-induced kinase 1; LC3, microtubule-associated protein 1 light chain 3; OPTN, optineurin; TRPML1, transient receptor potential mucolipin 1; TFEB, transcription factor EB; PGC-1α, peroxisome proliferator-activated receptor-γ coactivator 1α; NRF1, nuclear respiratory factor 1; TFAM, mitochondrial transcription factor A; PCBP2, poly(rC)-binding protein 2; TOM20, translocase of outer mitochondrial membrane 20; SFXN3, sideroflexin 3; VDAC1/2, voltage-dependent anion channel 1/2; 4-HNE, 4-hydroxynonenal; mPTP, mitochondrial permeability transition pore; ΔΨm, mitochondrial membrane potential; CoQ_10_, coenzyme Q_10_; ETC/OXPHOS, electron transport chain/oxidative phosphorylation; ATP, adenosine triphosphate; GSH, reduced glutathione; mtGPX4, mitochondrial glutathione peroxidase 4; PUFA, polyunsaturated fatty acid; PUFA-OOH, PUFA-containing lipid hydroperoxides; PUFA-OH, PUFA-containing lipid alcohol products; Fe^2+^, ferrous iron; H^+^, proton. Created in BioRender. sun, Y. (2026). https://BioRender.com/jlfpr0u, accessed on 7 June 2026.

Anti-apoptotic BCL-2 family members, including BCL-2, BCL-xL, and MCL-1, raise the mitochondrial stress threshold by restraining BAX/BAK-mediated outer mitochondrial membrane permeabilization and influencing mitochondrial Ca^2+^ stress and mPTP opening. Conversely, BCL-2 homology 3 (BH3) mimetics, including venetoclax (ABT-199), A-1331852, S63845, ABT-737, and obatoclax, remove this restraint and sensitize mitochondria to BAX/BAK-dependent mPTP opening [137]. In ferroptosis-related models, lipid-peroxidation-associated stress can intersect with apoptotic signaling, and BH3 mimetics may enhance, suppress, or redirect cell-death outcomes between ferroptosis and apoptosis in a context-dependent manner [138]. Thus, in PR ferroptosis-associated injury, the BCL-2-family axis may be viewed as a plausible mitochondrial node that modifies cell-death fate. A schematic synthesis of these mitochondrial homeostatic mechanisms is provided in Figure 3, and the evidence supporting each mitochondrial homeostatic axis is summarized in Table 1.

## 5. Imbalance in the RPE–PR Metabolic Ecosystem as a Trigger and Amplifier of PR Ferroptosis-Associated Injury

### 5.1. Impaired Outer Segment Phagocytosis: Disrupted Lipid Recycling and Iron Homeostasis

In aged mouse RPE, cell loss is accompanied by reduced phagosome-processing capacity, suggesting an age-related decline in post-phagocytic processing by the RPE [139]. Additional evidence for phagocytosis-related pathology comes from abnormalities in the Mer tyrosine kinase (MERTK) pathway. Pharmacological inhibition of MERTK leads to the accumulation of shed POS, increased phagosome and phagolysosome numbers within the RPE, delayed POS renewal, and early PR injury [140]. In models of MERTK deficiency, suppression of microglial activation delays retinal degeneration, suggesting that phagocytic failure is followed by secondary inflammatory injury [141]. Inadequate phagocytic processing also increases the burden on the RPE to degrade oxidatively modified outer-segment-derived substrates.

Krohne et al. found that uptake of 4-HNE- or MDA-modified POS promoted the accumulation of lipofuscin-like autofluorescent granules in RPE cells; this process was accompanied by lysosomal dysfunction and reduced autophagic activity [142]. Escrevente et al. showed that lipofuscin-like granules in the RPE may arise from phagosomes containing incompletely digested POS and that insufficient lysosomal acidification or reduced hydrolytic activity further exacerbated granule accumulation [143]. These findings indicate that impaired lysosomal degradation after phagocytosis contributes importantly to the persistent retention of POS-derived by-products. In addition, lipofuscin accumulation within the RPE can promote lysosomal membrane permeabilization and induce atypical necroptosis-like injury, thereby further exacerbating intracellular stress in RPE cells [144]. Such lysosomal stress may compromise the ability of the RPE to maintain outer-retinal homeostasis.

Beyond inflammatory amplification, insufficient phagocytic processing may also perturb redox and iron homeostasis within the RPE–PR functional unit. In sodium iodate models, ferritin in the RPE is associated with autophagy- and lysosome-related markers, and ferritin-containing vesicles can be released into the extracellular space, suggesting that, under stress conditions, the RPE may influence the local iron burden of neighboring outer-retinal cells through vesicle-associated pathways [145]. Therefore, the pathological consequences of defective outer-segment phagocytosis extend beyond prolonged retention of uncleared POS. Defective phagocytic processing also impairs the ability of the RPE to degrade, transport, and recycle outer-segment-derived lipids, thereby weakening the metabolic buffering capacity required to accommodate the increased lipid burden within the RPE–PR functional unit.

### 5.2. Disrupted Glucose–Lactate Partitioning and Weakening of Antioxidant Defenses Against Lipid Peroxidation

Glucose transport and lactate shuttling between the RPE and PRs together constitute a critical component of metabolic coupling in the outer retina. When this transcellular substrate partitioning is disrupted, the consequences extend beyond energy-supply imbalance; they also include impaired central carbon metabolism, which is required to sustain reducing power and protect PRs against lipid peroxidation. Lactate transport across the RPE–PR interface depends heavily on monocarboxylate transporters such as MCT1 and MCT3, as well as on their chaperone protein BSG; in *Bsg*-deficient models, maturation and membrane localization of the relevant MCTs are impaired, and this impairment is accompanied by metabolic disorganization in the outer retina and abnormal electroretinographic (ERG) responses [66].

Models of RPE mitochondrial dysfunction further support this view. In the RPE, impaired mitochondrial electron transport can induce pseudohypoxia, enhance aerobic glycolysis, and promote dedifferentiation, ultimately leading to structural and functional abnormalities in PRs. Activation of an alternative oxidase (AOX)-mediated electron transport bypass can partially reverse these metabolic abnormalities and ameliorate the associated retinal phenotypes [146]. Together with the glucose–lactate exchange framework discussed above, including direct in vivo isotope-tracing evidence [56], earlier biochemical evidence for a fuel-flexible retinal metabolic ecosystem [57], and review-level synthesis [58], these findings suggest that impaired RPE oxidative metabolism may weaken the substrate-partitioning pattern that normally helps preserve glucose availability for PRs. For PRs, the consequences of reduced glucose availability also extend beyond insufficient energy supply. PR-specific loss of glucose transporter 1 (GLUT1) leads to outer-segment shortening, reduced opsin levels in rods and cones, and ultimately impaired rod survival [60].

Consistent with this, simultaneous deletion of *HK2* and *PKM2* in rod photoreceptors causes early abnormalities in central glucose metabolism before overt cell loss becomes apparent. These abnormalities include reduced levels of the pentose phosphate pathway (PPP) intermediate sedoheptulose-7-phosphate (S7P) and are accompanied by thinning of the photoreceptor inner and outer segments, as well as thinning of the outer nuclear layer [64]. In addition, NADPH generated through the PPP contributes to GSH homeostasis in the retina [147]. Available evidence further indicates that GSH depletion markedly reduces the tolerance of PRs to lipid-peroxidation-associated injury [70]. Taken together, disruption of central carbon metabolism may indirectly weaken the antioxidant buffering capacity of PRs.

### 5.3. Impaired Retinaldehyde Clearance: atRAL and A2E-Driven Lipid Peroxidation and Ferroptosis-Associated Injury

Within PR outer-segment disc membranes, atRAL reversibly reacts with PE to form *N*-retinylidene-phosphatidylethanolamine (NRPE). When retinaldehyde handling or ATP-binding cassette subfamily A member 4 (ABCA4)-dependent transport is impaired, bisretinoid precursors and related lipid abnormalities may accumulate in the outer retina and RPE, increasing the burden of by-products such as *N*-retinylidene-*N*-retinylethanolamine (A2E) and contributing to Stargardt-like lipid dyshomeostasis [148,149]. Chen et al. showed that, in models of defective atRAL clearance, atRAL overload was accompanied by elevated Fe^2+^ levels, acyl-CoA synthetase long-chain family member 4 (ACSL4) upregulation, and suppression of xCT–GSH-dependent antioxidant defense, resulting in ROS accumulation, exacerbation of lipid peroxidation, and PR loss [20].

Additional studies have shown that atRAL promotes Fe^2+^ release by upregulating heme oxygenase-1 (HO-1), thereby exacerbating ROS-dependent lipid peroxidation [150]. c-Jun N-terminal kinase (JNK)/c-Jun/nuclear receptor coactivator 4 (NCOA4) signaling can further expand the labile iron pool by enhancing ferritinophagy, thereby aggravating atRAL-induced ferroptosis-associated injury [151]. Taken together, these findings suggest that defective atRAL clearance may not only increase lipid-peroxidation stress in PRs but also amplify ferroptosis-associated injury by disrupting iron homeostasis. Beyond the direct toxicity of atRAL to PRs, dysregulated retinaldehyde handling may also promote oxidative stress in the RPE by driving bisretinoid accumulation. In A2E-loaded RPE cells, blue-light exposure increases Fe^2+^ levels, depletes GSH, and suppresses the SLC7A11–GPX4 pathway in the RPE, and these changes are accompanied by canonical ferroptosis-associated features [152].

Consistent with this, mice with RPE-specific *Gpx4* deletion exhibit loss of RPE polarity, accumulation of lipid-peroxidation products, complement activation, and secondary PR loss [153]. These findings suggest that redox imbalance within the RPE may further amplify injury in the outer retina. Overall, timely atRAL clearance represents a visual-cycle–related retinoid-handling mechanism through which the PR–RPE unit limits the accumulation of phototransduction-derived retinoid by-products. When atRAL clearance fails, reactive retinoid intermediates are more likely to accumulate in both PRs and the RPE, thereby promoting oxidative stress and lipid-peroxidation-associated injury.

## 6. Therapeutic Strategies: Reducing the Biological Susceptibility of PRs to Ferroptosis-Associated Injury

### 6.1. Interrupting the Chain Reaction of Lipid Peroxidation

Among current experimental therapeutic strategies, direct interruption of the lipid-peroxidation cascade is a major approach for limiting ferroptosis-associated injury in PRs. In both bright-light-induced retinal injury models and models with defective atRAL clearance, Fer-1 attenuated ferroptosis-associated changes and alleviated PR degeneration [20,154], supporting the protective potential of chain-breaking interventions. However, blocking radical propagation alone may not be sufficient to fully suppress the injury cascade. Targeting upstream drivers of lipid peroxidation is therefore also therapeutically valuable. Consistent with this view, reducing mtROS can lessen the upstream oxidative pressure that drives lipid peroxidation.

In a recent study on atRAL-induced GSDME activation and mtROS-dependent photoreceptor ferroptosis, MitoTEMPO treatment reduced mtROS and intracellular ROS production in atRAL-loaded 661W PR-like cells, attenuated iron dyshomeostasis and lipid peroxidation, and alleviated photoreceptor ferroptosis and retinal degeneration in light-exposed *Abca4−/−Rdh8−/−* mice [155]. These findings suggest that limiting mitochondrial oxidative stress can partially attenuate ferroptosis-associated injury in atRAL-related models. Likewise, restricting iron-catalyzed oxidative reactions can blunt the upstream amplification of lipid peroxidation. In light-induced retinal injury models, deferiprone reduced photoreceptor death and attenuated oxidative-stress-related changes, including altered heme oxygenase 1 (Hmox1), ceruloplasmin (Cp), and complement component 3 (C3) expression, as well as nitrotyrosine levels [156].

In rd10 mice, the zinc–desferrioxamine complex (Zn–DFO) exerted stronger protective effects than Zn or DFO alone by improving electroretinographic responses, attenuating structural degeneration of PRs, and reducing oxidative-damage markers and ferritin-associated iron [157]. Taken together, these findings indicate that suppressing mitochondrial ROS and iron-driven oxidative stress can weaken the lipid-peroxidation cascade. Enhancing endogenous peroxide detoxification capacity is another key strategy. As discussed above, loss of mtGPX4 results in the accumulation of peroxidized DHA-containing PE species in the retina and progressive PR loss [39]. Maintenance of GPX4-dependent detoxification is therefore essential for PR homeostasis.

Consistent with this, inducible upregulation of GPX4 protected retinal structure and function in multiple oxidative injury models [158], whereas SLC7A11 overexpression suppressed light-induced ferroptosis-associated pathways and photoreceptor degeneration [17]. Strengthening peroxide detoxification via the xCT–GSH–GPX4 axis may thus represent an additional therapeutic approach to suppress lipid-peroxidation-associated injury. This axis also provides a mechanistic rationale for NAC, which can replenish intracellular cysteine availability, serve as a GSH precursor, and may support GPX4-mediated lipid hydroperoxide reduction by acting as a reducing substrate similar to cysteine [159]. In RP, oral NAC has been clinically evaluated in a phase I trial, where it was safe and well tolerated and suggested a possible improvement in macular cone function [160].

At the substrate level, reducing the oxidative susceptibility of PUFA-rich membrane lipids may further lower PR susceptibility to ferroptosis. Liu et al. showed that deuterated DHA suppressed the formation of the oxidative product carboxyethylpyrrole (CEP) in a model of retinal iron overload and markedly preserved outer-retinal structure and function [161]. This finding suggests that reducing the intrinsic reactivity of PUFA-rich membrane lipid substrates can limit the initiation and amplification of lipid-peroxidation chain reactions at the substrate level. Long-term Age-Related Eye Disease Study 2 (AREDS2) follow-up supports lutein and zeaxanthin as safer replacements for β-carotene in AMD-directed nutritional supplementation and suggests an association with reduced progression to late AMD, supporting the clinical relevance of carotenoid-based approaches [162]. In parallel, the soluble carotenoprotein AstaP has emerged as a protein-mediated carotenoid-delivery platform; AstaP–zeaxanthin scavenged ROS related to lipofuscin/bisretinoid photooxidation and suppressed oxidative stress in lipofuscin-enriched PR/RPE cell models, suggesting a preclinical strategy to improve carotenoid delivery or bioaccessibility in PR/RPE-relevant systems [163].

Several natural products have shown experimental therapeutic potential by simultaneously modulating iron homeostasis, redox status, and mitochondrial function. Evidence from oxidative-injury models, including H_2_O_2_-treated 661W cells and rd10 mice, shows that Fructus Lycii and Salvia miltiorrhiza extract (FSE) alleviates ferroptosis-associated injury in PRs and modulates the p53–SLC7A11–GPX4 signaling axis; Fer-1 produced similar antiferroptotic effects in these models [164]. Similarly, in 661W cells with atRAL accumulation, crocin attenuated oxidative stress, mitochondrial damage, Fe^2+^ accumulation, and lipid peroxidation, while also modulating the Kelch-like ECH-associated protein 1 (KEAP1)–NRF2–HO-1 pathway. Salvianic acid A (SAA), in turn, reduced iron deposition, lipid peroxidation, and mitochondrial damage in iron-overload models and modulated the expression of ferroptosis-related molecules, including ACSL4, GPX4, and SLC7A11 [22,165].

Overall, ferroptosis-associated PR injury may be mitigated not only by directly interrupting the lipid-peroxidation cascade but also by suppressing mitochondrial ROS and iron-driven oxidative stress, enhancing peroxide detoxification through the xCT–GSH–GPX4 axis, and reducing the intrinsic reactivity of oxidation-prone membrane lipid substrates. Together, these approaches may reduce PR vulnerability to lipid-peroxidation-associated injury.

### 6.2. Stabilizing Mitochondrial Redox Homeostasis, Dynamics, and Quality Control

Promoting the clearance of damaged mitochondria and limiting pathological fission may reduce PR susceptibility to ferroptosis-associated injury by preserving mitochondrial redox homeostasis. Among upstream regulators, AMP-activated protein kinase (AMPK) is a central kinase that links energy metabolism to mitochondrial homeostasis and coordinates mitochondrial biogenesis, dynamics, and mitophagy [166]. In models of acute light-induced injury and inherited retinal degeneration, metformin-mediated AMPK activation alleviated injury to PRs and the RPE, with concomitant reductions in oxidative stress and improvements in mitochondrial energy metabolism [167]. In high-glucose-treated 661W cells, AMPK activation improved mitochondrial membrane potential and morphology, increased mitochondrial DNA copy number, and upregulated PGC-1α–NRF1–TFAM signaling, and this was accompanied by increased LC3-II levels and reduced sequestosome 1 (p62/SQSTM1) levels [168].

Consistent with this, after bright-light-induced injury, the AMPK agonist 5-aminoimidazole-4-carboxamide ribonucleotide (AICAR) preserved retinal oxygen consumption, cytochrome c oxidase activity, and ATP levels while maintaining visual function and PR survival [169]. Beyond AMPK signaling, enhancing nicotinamide adenine dinucleotide (NAD^+^) metabolism provides an additional strategy for mitochondrial protection. Nicotinamide riboside (NR) produced sustained increases in retinal NAD^+^ levels, improved outer-retinal structure and function, and reduced apoptosis and inflammatory responses [170]. Nicotinamide mononucleotide (NMN), in turn, reduced infiltration by CD11b-positive inflammatory cells and PR death, decreased oxidative damage, preserved outer nuclear layer (ONL) thickness, and activated sirtuin 1 (SIRT1)–HO-1 signaling [171]. Taken together, these findings suggest that AMPK activation and NAD^+^ metabolic support help preserve mitochondrial respiration, ATP supply, and stress-response capacity, thereby indirectly enhancing PR tolerance to oxidative stress and potentially reducing susceptibility to lipid-peroxidation-associated injury.

From the standpoint of mitochondrial renewal and quality control, promoting mitochondrial biogenesis and enhancing the clearance of damaged mitochondria are also therapeutically relevant. The PGC-1α activator ZLN005 enhanced mitochondrial biogenesis, improved visual function, and preserved ONL thickness in rd1 mice [111]. In 661W cell models of oxidative injury, enhancement of PINK1/Parkin-mediated mitophagy and activation of the mitochondrial unfolded protein response (mtUPR) preserved mitochondrial membrane potential, supported mitochondrial quality control, and attenuated cellular injury [106]. In retinal detachment models, the TRPML1 agonist ML-SA1 also improved ONL structure and reduced PR apoptosis [172]. These findings indicate that strengthening mitochondrial renewal, mitophagy, and autophagy–lysosome-mediated clearance helps preserve mitochondrial quality-control capacity in PRs and may reduce their susceptibility to oxidative and lipid-peroxidation-associated injury. Stabilizing mitochondrial dynamics represents another feasible strategy.

Multiple models support the view that pharmacological or genetic interventions can limit pathological fission in PR mitochondria. Mdivi-1, commonly used to probe DRP1-related mitochondrial fission [173], can also reversibly inhibit complex I-linked respiration and modulate reverse-electron-transfer–mediated ROS production [174,175]. In type 1 diabetic retina and high-glucose-treated 661W PR-like cells, Mdivi-1 alleviated DRP1-related mitochondrial abnormalities, including swelling, fragmentation, cristae disruption, and membrane-potential loss, with improved retinal morphology and function [98]. Downregulation of microRNA-181a (miR-181a) and microRNA-181b (miR-181b) modulated DRP1 expression, reduced excessive mitochondrial fragmentation in rhodopsin P347S (RHO-347S) photoreceptors, and delayed the progression of inherited retinal degeneration [176]. In retinal detachment models, She et al. further showed that injury activated a ROS–DRP1-dependent mitochondrial fission pathway in PRs, whereas DRP1 inhibition preserved mitochondrial structure and alleviated PR damage [177].

Beyond fission control, mPTP-directed and mitochondrial membrane-stabilizing approaches suggest that mitochondrial permeability control can influence ferroptosis-associated injury. In a non-retinal C5b-9/IP3R-driven ferroptosis model, cyclosporin A, a classical inhibitor of CypD-dependent mPTP opening, preserved mitochondrial membrane potential and OXPHOS components and attenuated lipid ROS accumulation, Fe^2+^ accumulation, MDA production, ACSL4/COX-2 upregulation, and GSH/GPX4 loss [178]. At the inner membrane, elamipretide, a mitochondria-targeted cardiolipin-stabilizing peptide, was evaluated in the randomized, placebo-controlled phase II ReCLAIM-2 trial in patients with dry AMD and noncentral geographic atrophy; although the trial did not meet its primary endpoints, elamipretide was associated with slower ellipsoid-zone attenuation/loss, supporting its role as a mitochondrial membrane-stabilizing strategy [179]. At the outer mitochondrial membrane, VDAC1 oligomerization-targeting compounds, including NSC15364 and DIDS, suppressed mitochondrial ROS generation and cellular and mitochondrial lipid peroxidation in cysteine deprivation- and RSL3-induced ferroptosis models [130].

Overall, improving mitochondrial energy metabolism, enhancing mitochondrial renewal and quality control, and limiting pathological fission may collectively represent promising strategies for reducing PR susceptibility to oxidative stress and ferroptosis-associated lipid peroxidation. These interventions share a common feature: they enhance the tolerance of PRs to oxidative stress, membrane lipid peroxidation, and mitochondrial injury by stabilizing mitochondrial homeostasis.

### 6.3. Restoring RPE–PR Metabolic Coupling and Iron Homeostasis

Processing of phagocytosed POS and POS-derived lipid metabolism support RPE–PR metabolic coupling [55,180], and RPE65-dependent retinoid handling sustains visual-cycle function in the outer retina [34]. Together, these processes help maintain the metabolic and homeostatic relationship between the RPE and PRs. When this coupling is disrupted, PRs may experience abnormal substrate supply and increased oxidative stress. Accordingly, restoring post-phagocytic processing, oxidative metabolism, and local iron buffering in the RPE may provide an upstream strategy for reducing PR susceptibility to ferroptosis-associated injury. Restoration of post-phagocytic POS processing and phagosome maturation in the RPE may help re-establish lipid turnover and substrate allocation in the outer retina. One study showed that human fetal retinal pigment epithelium (hfRPE) and ARPE-19 cells exhibited increased β-hydroxybutyrate (β-HB) release following POS phagocytosis, whereas RPE explants from melanoregulin-deficient (*Mreg*−/−) and *Abca4*−/− mice displayed delayed peak β-HB release relative to peak phagocytic activity [52].

Consistent with this, aged mouse RPE displays reduced post-phagocytic processing capacity [139], whereas pharmacological suppression of mitochondrial fission improves POS phagocytosis in RPE models of aging-related mitochondrial dysfunction [181]. In Royal College of Surgeons (RCS) rats, RPE-targeted delivery of AAV8-Y733F-bestrophin 1 (BEST1)-human MERTK (hMERTK) conferred long-term retinal protection, further supporting the view that restoration of RPE phagocytic function helps maintain outer-retinal homeostasis [182]. Taken together, these findings indicate that improving post-phagocytic processing in the RPE may reduce the retention of POS-derived lipids and their oxidatively modified products in the outer retina, thereby reducing oxidative pressure in the microenvironment surrounding PRs.

The RPE–PR support framework also extends beyond POS clearance to visual-cycle restoration. In the RPE, RPE65 catalyzes the conversion of all-trans-retinyl esters into 11-cis-retinol, which is subsequently oxidized to the 11-cis-retinal chromophore required for photoreceptor visual pigment regeneration. Accordingly, AAV2-mediated *RPE65* gene augmentation with voretigene neparvovec-rzyl is best positioned as an RPE-directed strategy that restores visual-cycle support upstream of secondary photoreceptor dysfunction in patients with confirmed biallelic RPE65 mutation-associated retinal dystrophy and sufficient viable retina [34,183]. Longer-term follow-up supports this mechanism, showing sustained improvements in functional vision, light sensitivity, and visual field measures in patients with biallelic RPE65-mediated inherited retinal disease [184].

In addition, restoring oxidative metabolism and strengthening antioxidant defenses against lipid peroxidation in the RPE may provide another means of supporting PR metabolism. In models of RPE electron transport chain (ETC) deficiency, RPE-specific expression of AOX partially reversed pseudohypoxia-associated metabolic reprogramming and differentiation defects, improved cellular stress responses, and preserved retinal structure and function [146]. Consistent with this, in models with RPE-specific *Gpx4* deletion, lipid peroxidation products such as acrolein, MDA, and 4-HNE accumulated in the RPE, and pharmacological suppression of lipid peroxidation-related injury, such as Fer-1 or α-tocopherol treatment, alleviated RPE degeneration and secondary PR loss [153]. Strengthening defenses against lipid peroxidation in the RPE may therefore protect the RPE itself while indirectly reducing PR exposure to oxidative stress and the risk of ferroptosis-associated injury.

Beyond restoring metabolic support, enhancing iron buffering and iron transport in the RPE may also help prevent local iron dyshomeostasis from affecting PRs. Intraocular iron injection induces PR death and oxidative injury in the outer retina and subsequently elicits geographic atrophy (GA)-like changes [185], underscoring the sensitivity of the outer retina to disturbed iron homeostasis. Consistent with this, aqueous humor samples from patients with early GA show elevated iron levels and reduced transferrin iron-binding capacity. In induced pluripotent stem cell (iPSC)-derived RPE models, transferrin (TF) supplementation improved iron homeostasis and attenuated oxidative stress, mitochondrial injury, and ferroptosis-associated changes [186]. Moreover, TF-based non-viral gene therapy further supports iron buffering as a retinal-protective strategy, because sustained intraocular TF production delayed structural and functional degeneration in the RCS rat model of retinitis pigmentosa and reduced ocular malondialdehyde levels [187].

Neurotrophic approaches can also be incorporated into the RPE–PR support framework. Revakinagene taroretcel-lwey, formerly known as NT-501, is an intraocular encapsulated cell therapy for intravitreal implantation, consisting of a semipermeable hollow-fiber capsule loaded with the human RPE cell line NTC-201-6A, which expresses recombinant human ciliary neurotrophic factor (rhCNTF), thereby enabling sustained intraocular CNTF delivery [188]. In two pivotal phase 3 trials involving patients with macular telangiectasia type 2, revakinagene taroretcel significantly slowed ellipsoid-zone area loss compared with sham treatment, supporting encapsulated CNTF delivery as a clinically validated neurotrophic strategy for slowing macular PR loss in this disease context [189]. Taken together, these findings suggest that therapeutic strategies directed at the RPE–PR unit—including restoration of POS-processing capacity, visual-cycle retinoid handling, RPE oxidative metabolism, local iron-buffering capacity, and CNTF-based trophic signaling—may help reduce the pro-oxidative microenvironment surrounding PRs and thereby lower their vulnerability to ferroptosis-associated injury [41,190]. The therapeutic strategies discussed in Section 6.1, Section 6.2 and Section 6.3 are summarized in Table 2.

## 7. Conclusions and Perspectives

This review frames PR vulnerability to ferroptosis-associated injury as a multilayered biological state shaped by membrane lipid substrates, mitochondrial homeostasis, and the RPE–PR microenvironment. It highlights the DHA-enriched lipid composition of PR outer segments, the chronically high metabolic demand and spatially specialized mitochondrial organization of PRs, RPE–PR metabolic coupling sustained by outer-segment renewal, phagocytic turnover, glucose transport, lactate shuttling, and visual-cycle–related atRAL clearance and bisretinoid handling, as well as lipid-peroxidation defense systems centered on the xCT–GSH–GPX4 axis and mtGPX4. Disturbances in mitochondrial dynamics, quality control, and biogenesis, together with iron dyshomeostasis and disruption of the RPE–PR unit, may compromise both PR-intrinsic homeostasis and tissue-level metabolic support. In this context, impaired atRAL clearance and A2E/bisretinoid accumulation provide an important link between visual-cycle stress, iron dyshomeostasis, and lipid peroxidation. Together, these considerations support the central implication of this review: ferroptosis-associated injury in PRs should be regarded not as an isolated terminal cell-death event, but as a state of outer-retinal metabolic and redox decompensation arising from the convergence of lipid-substrate burden, mitochondrial instability, and impaired RPE–PR support.

Ferroptosis-related mechanisms may offer a cross-disease lens for interpreting convergent features of PR injury in AMD, DR, inherited retinal degeneration, light-induced retinal injury, retinal detachment, and visual-cycle-related disorders, although the strength of evidence varies across models and disease contexts. Clinically, this framework is relevant because PR structural abnormalities can increasingly be measured before end-stage retinal atrophy. Large-scale OCT analysis has identified photoreceptor segment thinning as an early biomarker associated with future AMD risk, and quantitative optical coherence tomography (OCT) has revealed rod-predominant photoreceptor abnormalities in early diabetic retinopathy [191,192]. These observations do not, by themselves, establish ferroptosis as the operative mechanism in patients, but they indicate that PR-centered injury can be captured at clinically accessible stages. Future translational studies should therefore integrate OCT, adaptive optics imaging, optoretinography, ERG, and microperimetry phenotypes with molecular readouts of iron load, lipid peroxidation, GSH–GPX4 capacity, retinoid stress, mitochondrial dysfunction, and RPE metabolic failure.

Rod–cone heterogeneity should also be considered when interpreting PR susceptibility to ferroptosis-associated injury. This distinction is clinically relevant because AMD often shows early involvement of photoreceptors in parafoveal macular regions where rods predominate, whereas RP is generally characterized by primary rod degeneration followed by secondary cone loss [193,194]. Rod-dominant samples contain higher levels of PUFAs, including LC- and VLC-PUFAs, whereas cone-enriched regions show higher pyruvate metabolism and tricarboxylic acid cycle activity. These differences suggest that rods may face greater substrate-driven lipid-peroxidation pressure, whereas cone vulnerability may be more closely shaped by mitochondrial energy metabolism and redox resilience. Single-cell transcriptomic evidence from the rd10 RP model further reveals early transcriptional responses associated with rod degeneration and secondary cone involvement, reinforcing the need to resolve PR responses at cell-type and subtype levels [195].

However, direct comparative evidence for rod- versus cone-specific ferroptosis susceptibility remains limited. Future studies should therefore distinguish rod- and cone-specific vulnerabilities and avoid treating photoreceptors as a homogeneous cell population. A further limitation concerns evidence derived from the 661W cell line. Although 661W cells are widely used as an immortalized PR-like retinal cell model, their cellular identity remains debated, and they have been reported to show properties of both retinal ganglion precursor-like cells and photoreceptor cells [71,72]. This evidence hierarchy is important for translation because ferroptosis-targeted strategies require validation in models that retain PR subtype identity, outer-segment renewal, RPE–PR coupling, and disease-stage-specific outer-retinal architecture. Therefore, conclusions derived from 661W-based models in this review are interpreted as supportive and hypothesis-generating rather than definitive primary-PR evidence, particularly when not accompanied by primary photoreceptor, retinal organoid, or in vivo PR-specific validation.

From a translational perspective, future interventions may need to extend beyond downstream lipid-radical scavenging and instead be developed as combination strategies that reduce PR susceptibility to ferroptosis-associated injury. This may involve limiting iron-catalyzed oxidative reactions, strengthening peroxide detoxification through the xCT–GSH–GPX4 axis and mtGPX4-dependent defenses, and reducing the reactivity of PUFA-rich membrane lipid substrates. Recent preclinical evidence supports the translational relevance of iron-handling and lipid-redox ferroptosis axes: deferiprone protected photoreceptors by inhibiting ferroptosis after experimental retinal detachment [196], whereas puerarin and astragaloside IV reduced iron-overload-induced retinal ferroptotic injury through mechanisms related to Nrf2 signaling and iron handling [197,198]. Early clinical evidence from a phase I trial of oral N-acetylcysteine in RP, together with subsequent locus-level analysis, further suggests that redox-supportive approaches can be linked to measurable patient-level functional outcomes, including macular sensitivity [160,199]. Together, these findings support the translational plausibility of ferroptosis-related interventions, while indicating that patient selection, therapeutic timing, retinal delivery, and long-term safety remain unresolved.

RPE-directed strategies may also be needed to reduce PR vulnerability. Stabilizing post-phagocytic POS processing, retinoid clearance, mitochondrial quality control, local iron buffering, and glucose–lactate/ketone metabolic support in the RPE may decrease upstream stress imposed on PRs. In this regard, Fer-1 mitigates atRAL-induced RPE ferroptosis and subsequent RPE injury and photoreceptor degeneration in *Abca4−/−Rdh8−/−* models, reinforcing the idea that ferroptosis-related injury can propagate through the RPE–PR unit [200]. Thus, optimal therapy may require temporally staged or combined targeting of PR-intrinsic lipid-redox defenses, mitochondrial homeostasis, and RPE-mediated metabolic support. Key unresolved questions include when ferroptosis-associated molecular changes become irreversible, which biomarkers best identify a treatable ferroptosis-susceptible state, whether rods and cones require different protective strategies, and whether RPE-first or PR-first intervention is preferable in different retinal diseases.

Overall, the framework proposed here provides a conceptual and translational basis for developing PR-protective strategies for blinding retinal diseases, including DR, AMD, and RP. Its main value is to shift attention from ferroptosis as a downstream cell-death label toward ferroptosis susceptibility as a modifiable outer-retinal vulnerability state. This perspective may help guide future work toward early-stage biomarkers, disease-stage-specific therapeutic windows, and rational combination approaches that preserve, as far as possible, the membrane lipid composition and metabolic specialization required for visual function.

## Figures and Tables

**Table 1:** Evidence-graded mitochondrial homeostatic axes regulating photoreceptor susceptibility to ferroptosis-associated injury.

Mechanistic Axis	Key Mechanism	Main Supporting Model	Evidence Level	Ref.
mtGPX4-dependent phospholipid detoxification	mtGPX4 detoxifies phospholipid hydroperoxides. PR-specific *Gpx4* loss causes rapid degeneration with outer-segment, ciliary, and mitochondrial defects; mtGPX4 loss increases peroxidized DHA-containing PE and promotes cone–rod loss.	PR-specific *Gpx4* knockout mice; mtGPX4 knockout mice	A	[38,39]
OPA1/mitofusin-dependent fusion and PR mitochondrial architecture	Mitochondrial fusion maintains rod PR morphology and metabolism. OPA1 loss disrupts inner-segment mitochondrial alignment and inner-membrane organization.	Rod PR mitofusin models; PR OPA1 models; OPA1 structural studies	B	[50,89,90]
MFN2–MERC/IP3R Ca^2+^ coupling	MFN2 maintains MERC structure; disruption of MERC/IP3R-mediated Ca^2+^ handling may alter mitochondrial ROS/lipid homeostasis and contribute to hypoxic PR injury; SH-SY5Y neuroblastoma-cell ferroptosis data support IP3R-mediated mitochondrial Ca^2+^ overload.	MFN2/MERC models; hypoxic PR injury; SH-SY5Y neuroblastoma-cell ferroptosis model	B	[91,92,93,94]
DRP1-dependent mitochondrial fission	DRP1 activation and mitochondrial fragmentation accompany ferroptosis in NIH-3T3/HT-1080/H441/A549 cells, *Drp1^fl/fl^* MEFs, and HT22 neurons; diabetic retinas and high-glucose-treated 661W PR-like cells show p-DRP1, fragmentation, cristae disruption, and membrane-potential loss.	NIH-3T3/HT-1080/H441/A549 cells, *Drp1^fl/fl^* MEFs, and HT22 neurons; T1DM-NDR retina; high-glucose 661W cells; AMD donor RPE	B	[95,96,97,98,99]
PINK1/Parkin and TRPML1–TFEB/OPTN quality control	Mitophagy and lysosomal biogenesis remove damaged mitochondria. Evidence from the developing retina, H_2_O_2_-treated 661W PR-like cells, and RPE models supports this quality-control axis.	Developing retina; H_2_O_2_-treated 661W cells; *PGC-1α/NFE2L2*-deficient and phagocytic RPE models	B	[103,104,105,106,107,108,109]
PGC-1α/NRF1/TFAM mitochondrial biogenesis	Mitochondrial biogenesis supports PR energy metabolism. PGC-1α activation protects rd1/P23H models; rod *Nrf1* loss causes mitochondrial dysfunction and PR degeneration; RPE biogenesis defects may weaken PR support.	rd1 and P23H RP models; rod-specific *Nrf1* models; RPE biogenesis/metabolism studies	B	[110,111,112,113,114,115]
TSPO-related retinal stress axis	TSPO is enriched in reactive retinal microglia and can participate in photoreceptor-debris-driven TSPO–NOX1 oxidative responses. TSPO ligands or microglial TSPO deletion reduce inflammatory/oxidative injury in retinal or PR-relevant models, but the link to PR ferroptosis remains indirect.	Retinal degeneration/reactive microglia models; TSPO–NOX1 phagocyte model; 661W/rd10 TSPO-ligand studies	B	[132,133,134,135]
cGAS–STING inflammatory amplification	Retinal oxidative stress is associated with cGAS–STING activation, damaged-DNA leakage in PRs, and inflammatory PR degeneration; DNA-induced cGAS-dependent microglial activation is also linked to PR degeneration. However, cGAS–STING-mediated PR ferroptosis remains unproven, and the cGAS–DRP1–ferroptosis link is mainly based on non-retinal cancer-cell models.	Oxidative stress-induced retinal degeneration; DNA-induced microglial activation/PR degeneration models; non-retinal cGAS–DRP1 ferroptosis models	C	[100,101,102]
PCBP2–TOM20–SFXN3 mitochondrial iron entry	KU812/K562 leukemia-cell data identify this axis as a mitochondrial iron-entry route and RSL3-sensitivity modifier. *Sfxn3* mutations cause progressive outer-retinal degeneration in mice.	KU812/K562 leukemia-cell mitochondrial iron-entry models; *Sfxn3* mutant retina	C	[121,122]
Cardiolipin/CoQ inner-membrane lipid homeostasis	Cardiolipin and CoQ support respiratory-chain supercomplex organization, respiration, and ATP production; cardiolipin-lacking yeast and PCBP1-depleted mouse-liver models link CL/CoQ depletion to bioenergetic impairment.	Cardiolipin-lacking yeast respiratory-supercomplex model; PCBP1-depleted mouse-liver model	C	[123,124]
mPTP/VDAC1 membrane-stabilizing axis	mPTP opening and VDAC1 oligomerization can connect mitochondrial permeability, membrane-potential loss, mtROS generation, GSH depletion, and lipid peroxidation. VDAC1-oligomerization inhibitors suppress ferroptosis-related mitochondrial injury in non-PR cells, while retinal VDAC1 data are supportive but not PR-specific.	VDAC1 oligomerization ferroptosis models; retinal ischemia–reperfusion model	C	[128,129,130,131]
BCL-2-mediated cell-death axis	BCL-2-family proteins modulate the mitochondrial stress threshold by restraining BAX/BAK-dependent outer-membrane permeabilization, Ca^2+^ stress, and mPTP opening. BH3 mimetics can alter ferroptosis–apoptosis crosstalk in a context-dependent manner; PR-specific ferroptosis evidence is not yet established.	BCL-2/BH3 mitochondrial-stress models; ferroptosis–apoptosis crosstalk models	C	[137,138]

Note: Evidence level A indicates direct photoreceptor/retinal genetic or pharmacological evidence linking the axis to ferroptosis- or lipid-peroxidation-associated photoreceptor degeneration; B indicates photoreceptor/retinal or outer-retinal evidence for mitochondrial/homeostatic injury with an indirect ferroptosis link or partial support from non-retinal ferroptosis models; C indicates mechanisms supported mainly by non-retinal studies or indirect retinal observations and requiring further photoreceptor-specific validation.

**Table 2:** Therapeutic strategies for reducing photoreceptor susceptibility to ferroptosis-associated injury.

Strategy	Targeted Vulnerability	Intervention Nodes	Expected PR/RPE Effect	Ref.
Lipid-peroxidation blockade	DHA/PUFA-rich outer-segment membranes; labile Fe^2+^; mtROS-driven initiation; limited cysteine/GSH supply; impaired xCT–GSH–GPX4 detoxification; oxidation-prone lipid substrates.	Fer-1/α-tocopherol; MitoTEMPO; deferiprone/Zn–DFO; NAC-supported cysteine/GSH replenishment; SLC7A11/GPX4 support; deuterated DHA; lutein/zeaxanthin; AstaP–zeaxanthin; FSE, crocin, and SAA as multitarget redox/iron modulators.	Limits lipid-radical propagation, Fe^2+^/mtROS-driven amplification, and PUFA oxidizability; enhances cysteine/GSH availability, phospholipid-hydroperoxide detoxification, and carotenoid-related antioxidant support.	[17,20,22,39,153,154,155,156,157,158,159,160,161,162,163,164,165]
Mitochondrial redox and quality control	High PR energy demand; mtROS leakage; ATP insufficiency; defective biogenesis/mitophagy and autophagy–lysosome clearance; excessive DRP1-dependent fission; mPTP/VDAC1-related membrane destabilization.	AMPK activation with metformin/AICAR; NAD^+^ support with NR/NMN; PGC-1α–NRF1–TFAM activation with ZLN005; PINK1/Parkin and mtUPR activation; TRPML1–lysosomal support; DRP1 inhibition or *miR-181a/b* downregulation; cyclosporin A; elamipretide; VDAC1 oligomerization inhibitors (NSC15364/DIDS).	Preserves respiration, membrane potential, ATP supply, and antioxidant buffering; promotes mitochondrial renewal and clearance; reduces fission-associated mtROS and lipid-peroxidation pressure; stabilizes mPTP/cardiolipin/VDAC1-linked membrane homeostasis.	[98,106,111,128,130,166,167,168,169,170,171,172,173,174,175,176,177,178,179]
RPE–PR metabolic support and iron buffering	RPE post-phagocytic POS-processing failure; oxidized POS/lipofuscin burden; disrupted glucose–lactate/ketone support; RPE65/visual-cycle insufficiency; reduced CNTF trophic support; RPE oxidative/ferroptotic stress; reduced local iron buffering.	MERTK-directed restoration of POS phagocytosis/phagosome maturation; post-phagocytic processing support; RPE mitochondrial dynamics modulation; AOX-mediated rescue of RPE ETC dysfunction; Fer-1-mediated RPE lipid-peroxidation suppression; voretigene neparvovec-rzyl; TF supplementation/non-viral TF gene therapy; revakinagene taroretcel/CNTF.	Improves lipid recycling/substrate allocation; limits oxidized POS-derived lipids; restores visual-cycle support; provides sustained CNTF trophic signaling; strengthens RPE antioxidant support; buffers labile iron and reduces secondary oxidative pressure on PRs.	[52,139,146,153,180,181,182,183,184,185,186,187,188,189,190]

## Data Availability

No new data were created or analyzed in this study. Data sharing is not applicable to this article.

## Abbreviations

The following abbreviations are used in this manuscript:

4-HNE	4-hydroxynonenal
A2E	N-retinylidene-N-retinylethanolamine
AMD	age-related macular degeneration
AMPK	AMP-activated protein kinase
AOX	alternative oxidase
atRAL	all-trans-retinal
BH_4_	tetrahydrobiopterin
cGAS	cyclic GMP–AMP synthase
CL	cardiolipin
CoQ_10_	coenzyme Q_10_
DHA	docosahexaenoic acid
DHODH	dihydroorotate dehydrogenase
DR	diabetic retinopathy
DRP1/*Drp1*	dynamin-related protein 1
ETC	electron transport chain
Fer-1	ferrostatin-1
FSP1	ferroptosis suppressor protein 1
GA	geographic atrophy
GCH1	GTP cyclohydrolase 1
GPX4/*Gpx4*	glutathione peroxidase 4
GSH	glutathione
HO-1	heme oxygenase-1
IP3R	inositol 1,4,5-trisphosphate receptor
LC- and VLC-PUFAs	long-chain and very-long-chain polyunsaturated fatty acids
LC3/LC3B	microtubule-associated protein 1 light chain 3/light chain 3 beta
MCT1/MCT3/MCTs	monocarboxylate transporter 1/3/(s)
MDA	malondialdehyde
MERC/MERCs	mitochondria–endoplasmic reticulum contact site(s)
MFN2	mitofusin 2
mtGPX4	mitochondrial glutathione peroxidase 4
mtROS	mitochondrial reactive oxygen species
mtUPR	mitochondrial unfolded protein response
NCOA4	nuclear receptor coactivator 4
*NFE2L2*/NRF2	nuclear factor erythroid 2-related factor 2 gene/protein
NRF1/*Nrf1*	nuclear respiratory factor 1
ONL	outer nuclear layer
OPA1	optic atrophy 1
OPTN	optineurin
PCBP2	poly(rC)-binding protein 2
PE	phosphatidylethanolamine
PGC-1α	peroxisome proliferator-activated receptor gamma coactivator 1-alpha
PINK1	PTEN-induced kinase 1
POS	photoreceptor outer segment(s)
PR	photoreceptor
PUFA/PUFAs	polyunsaturated fatty acid(s)
ROS	reactive oxygen species
RP	retinitis pigmentosa
RPE	retinal pigment epithelium
RPE65	retinal pigment epithelium-specific 65 kDa protein
RPE–PR	retinal pigment epithelium–photoreceptor
RSL3	RAS-selective lethal 3
SLC7A11	solute carrier family 7 member 11
STING	stimulator of interferon genes
TFAM	mitochondrial transcription factor A
TOM20	translocase of outer mitochondrial membrane 20
VDAC1/VDAC2	voltage-dependent anion channel 1/2
VEGF	vascular endothelial growth factor
xCT	cystine/glutamate antiporter

## References

[1] Guymer R.H., Campbell T.G. (2023). Age-related macular degeneration. Lancet.

[2] GBD 2021 Global AMD Collaborators (2025). Global burden of vision impairment due to age-related macular degeneration, 1990–2021, with forecasts to 2050: A systematic analysis for the Global Burden of Disease Study 2021. Lancet Glob. Health.

[3] Fleckenstein M., Schmitz-Valckenberg S., Chakravarthy U. (2024). Age-Related Macular Degeneration: A Review. JAMA.

[4] Liu Y., Zong X., Cao W., Zhang W., Zhang N., Yang N. (2024). Gene Therapy for Retinitis Pigmentosa: Current Challenges and New Progress. Biomolecules.

[5] Murati Calderón R.A., Emanuelli A., Izquierdo N. (2025). Retinitis Pigmentosa: From Genetic Insights to Innovative Therapeutic Approaches—A Literature Review. Medicina.

[6] Yang M., So K.-F., Lam W.-C., Lo A.C.Y. (2021). Cell Ferroptosis: New Mechanism and New Hope for Retinitis Pigmentosa. Cells.

[7] Teo Z.L., Tham Y.-C., Yu M., Chee M.L., Rim T.H., Cheung N., Bikbov M.M., Wang Y.X., Tang Y., Lu Y. (2021). Global Prevalence of Diabetic Retinopathy and Projection of Burden through 2045: Systematic Review and Meta-analysis. Ophthalmology.

[8] Rajagopal R., Kern T. (2025). Clinical Evidence of a Photoreceptor Origin in Diabetic Retinal Disease. Ophthalmol. Sci..

[9] Tonade D., Kern T.S. (2021). Photoreceptor cells and RPE contribute to the development of diabetic retinopathy. Prog. Retin. Eye Res..

[10] Sachdeva M.M. (2021). Retinal Neurodegeneration in Diabetes: An Emerging Concept in Diabetic Retinopathy. Curr. Diabetes Rep..

[11] Moraru A.D., Danielescu C., Iorga R.E., Moraru R.L., Zemba M., Branisteanu D.C. (2024). Review of Guideline Recommendations for Optimal Anti-VEGF Therapy in Age-Related Macular Degeneration. Life.

[12] Cheng S., Zhang S., Huang M., Liu Y., Zou X., Chen X., Zhang Z. (2024). Treatment of neovascular age-related macular degeneration with anti-vascular endothelial growth factor drugs: Progress from mechanisms to clinical applications. Front. Med..

[13] Samanta A., Aziz A.A., Jhingan M., Singh S.R., Khanani A.M., Chhablani J. (2021). Emerging therapies in nonexudative age-related macular degeneration in 2020. Asia-Pac. J. Ophthalmol..

[14] Liu W., Liu S., Li P., Yao K. (2022). Retinitis Pigmentosa: Progress in Molecular Pathology and Biotherapeutical Strategies. Int. J. Mol. Sci..

[15] Dixon S.J., Lemberg K.M., Lamprecht M.R., Skouta R., Zaitsev E.M., Gleason C.E., Patel D.N., Bauer A.J., Cantley A.M., Yang W.S. (2012). Ferroptosis: An iron-dependent form of nonapoptotic cell death. Cell.

[16] Yang Y., Lin Y., Han Z., Wang B., Zheng W., Wei L. (2024). Ferroptosis: A novel mechanism of cell death in ophthalmic conditions. Front. Immunol..

[17] Huang X., Zhang Y., Jiang Y., Li T., Yang S., Wang Y., Yu B., Zhou M., Zhang G., Zhao X. (2026). Contribution of ferroptosis and SLC7A11 to light-induced photoreceptor degeneration. Neural Regen. Res..

[18] Gao S., Gao S., Wang Y., Li N., Yang Z., Yao H., Chen Y., Cheng Y., Zhong Y., Shen X. (2023). Inhibition of Ferroptosis Ameliorates Photoreceptor Degeneration in Experimental Diabetic Mice. Int. J. Mol. Sci..

[19] Weiss M.E., Parrales P.E., Datta M., Fleishaker M., Dvoriantchikova G., Ivanov D., Hackam A.S. (2025). Identifying a role for oxytosis/ferroptosis in Pde6b-associated retinitis pigmentosa. Exp. Eye Res..

[20] Chen C., Chen J., Wang Y., Liu Z., Wu Y. (2021). Ferroptosis drives photoreceptor degeneration in mice with defects in all-trans-retinal clearance. J. Biol. Chem..

[21] Newton F., Megaw R. (2020). Mechanisms of photoreceptor death in retinitis pigmentosa. Genes.

[22] Yang B., Yang K., Chen J., Wu Y. (2024). Crocin protects the 661W murine photoreceptor cell line against the toxic effects of all-trans-retinal. Int. J. Mol. Sci..

[23] Guo M., Zhu Y., Shi Y., Meng X., Dong X., Zhang H., Wang X., Du M., Yan H. (2022). Inhibition of ferroptosis promotes retina ganglion cell survival in experimental optic neuropathies. Redox Biol..

[24] Swinkels D., Baes M. (2023). The essential role of docosahexaenoic acid and its derivatives for retinal integrity. Pharmacol. Ther..

[25] Agbaga M.P., Merriman D.K., Brush R.S., Lydic T.A., Conley S.M., Naash M.I., Jackson S., Woods A.S., Reid G.E., Busik J.V. (2018). Differential composition of DHA and very-long-chain PUFAs in rod and cone photoreceptors. J. Lipid Res..

[26] Longoni B., Demontis G.C. (2023). Polyunsaturated lipids in the light-exposed and prooxidant retinal environment. Antioxidants.

[27] Li B., Zhang T., Liu W., Wang Y., Xu R., Zeng S., Zhang R., Zhu S., Gillies M.C., Zhu L. (2020). Metabolic features of mouse and human retinas: Rods versus cones, macula versus periphery, retina versus RPE. iScience.

[28] Ball J.M., Chen S., Li W. (2022). Mitochondria in cone photoreceptors act as microlenses to enhance photon delivery and confer directional sensitivity to light. Sci. Adv..

[29] Hayes M.J., Tracey-White D., Hoh Kam J., Powner M.B., Jeffery G. (2021). The 3D organisation of mitochondria in primate photoreceptors. Sci. Rep..

[30] Lewis T.R., Klementieva N.V., Phan S., Castillo C.M., Kim K.-Y., Cao L.Y., Ellisman M.H., Arshavsky V.Y., Alekseev O. (2025). Unique ultrastructural organization of human rod photoreceptors. Commun. Biol..

[31] Caceres P.S., Rodriguez-Boulan E. (2020). Retinal pigment epithelium polarity in health and blinding diseases. Curr. Opin. Cell Biol..

[32] Kwon W., Freeman S.A. (2020). Phagocytosis by the retinal pigment epithelium: Recognition, resolution, recycling. Front. Immunol..

[33] Lewandowski D., Sander C.L., Tworak A., Gao F., Xu Q., Skowronska-Krawczyk D. (2022). Dynamic lipid turnover in photoreceptors and retinal pigment epithelium throughout life. Prog. Retin. Eye Res..

[34] Kiser P.D. (2022). Retinal pigment epithelium 65 kDa protein (RPE65): An update. Prog. Retin. Eye Res..

[35] Koppula P., Zhuang L., Gan B. (2021). Cystine transporter SLC7A11/xCT in cancer: Ferroptosis, nutrient dependency, and cancer therapy. Protein Cell.

[36] Rochette L., Dogon G., Rigal E., Zeller M., Cottin Y., Vergely C. (2023). Lipid Peroxidation and Iron Metabolism: Two Corner Stones in the Homeostasis Control of Ferroptosis. Int. J. Mol. Sci..

[37] Liu Y., Wan Y., Jiang Y., Zhang L., Cheng W. (2023). GPX4: The hub of lipid oxidation, ferroptosis, disease and treatment. Biochim. Biophys. Acta Rev. Cancer.

[38] Ueta T., Inoue T., Furukawa T., Tamaki Y., Nakagawa Y., Imai H., Yanagi Y. (2012). Glutathione peroxidase 4 is required for maturation of photoreceptor cells. J. Biol. Chem..

[39] Azuma K., Koumura T., Iwamoto R., Matsuoka M., Terauchi R., Yasuda S., Shiraya T., Watanabe S., Aihara M., Imai H. (2022). Mitochondrial glutathione peroxidase 4 is indispensable for photoreceptor development and survival in mice. J. Biol. Chem..

[40] Zhu G., Lin Y., Han Z., Cao H., Zhu K., Shi R., Deng Y., Li S., Yang Q., Lu X. (2026). Ferroptosis and the eye: Bridging the gap between cell death and vision preservation. Front. Immunol..

[41] Dohl J., Burns G., Singh M. (2025). The intersection of mitochondria, lipids, and ferroptosis: A new avenue for dry age-related macular degeneration. Apoptosis.

[42] Liu D., Liu Z., Liao H., Chen Z.-S., Qin B. (2024). Ferroptosis as a potential therapeutic target for age-related macular degeneration. Drug Discov. Today.

[43] Wang Y., Becker S., Finkelstein S., Dyka F.M., Liu H., Eminhizer M., Hao Y., Brush R.S., Spencer W.J., Arshavsky V.Y. (2024). Acyl-CoA synthetase 6 controls rod photoreceptor function and survival by shaping the phospholipid composition of retinal membranes. Commun. Biol..

[44] Swinkels D., Kocherlakota S., Das Y., Dane A.D., Wever E.J.M., Vaz F.M., Bazan N.G., Van Veldhoven P.P., Baes M. (2023). DHA shortage causes the early degeneration of photoreceptors and RPE in mice with peroxisomal β-oxidation deficiency. Investig. Ophthalmol. Vis. Sci..

[45] Shindou H., Koso H., Sasaki J., Nakanishi H., Sagara H., Nakagawa K.M., Takahashi Y., Hishikawa D., Iizuka-Hishikawa Y., Tokumasu F. (2017). Docosahexaenoic acid preserves visual function by maintaining correct disc morphology in retinal photoreceptor cells. J. Biol. Chem..

[46] Salas-Estrada L.A., Leioatts N., Romo T.D., Grossfield A. (2018). Lipids Alter Rhodopsin Function via Ligand-like and Solvent-like Interactions. Biophys. J..

[47] Kiel C., Prins S., Foss A.J.E., Luthert P.J. (2025). Energetics of the outer retina II: Calculation of a spatio-temporal energy budget in retinal pigment epithelium and photoreceptor cells based on quantification of cellular processes. PLoS ONE.

[48] Thoreson W.B. (2021). Transmission at rod and cone ribbon synapses in the retina. Pflug. Arch..

[49] Subramanya S., Goswami M.T., Miller N., Weh E., Chaudhury S., Zhang L., Andren A., Hager H., Weh K.M., Lyssiotis C.A. (2023). Rod photoreceptor-specific deletion of cytosolic aspartate aminotransferase, GOT1, causes retinal degeneration. Front. Ophthalmol..

[50] Meschede I.P., Ovenden N.C., Seabra M.C., Futter C.E., Votruba M., Cheetham M.E., Burgoyne T. (2020). Symmetric arrangement of mitochondria: Plasma membrane contacts between adjacent photoreceptor cells regulated by Opa1. Proc. Natl. Acad. Sci. USA.

[51] Milićević N., Mazzaro N., de Bruin I., Wils E., ten Brink J.B., ten Asbroek A.L.M.A., Mendoza J., Bergen A.A., Felder-Schmittbuhl M.-P. (2019). Rev-Erbα and Photoreceptor Outer Segments modulate the Circadian Clock in Retinal Pigment Epithelial Cells. Sci. Rep..

[52] Reyes-Reveles J., Dhingra A., Alexander D., Bragin A., Philp N.J., Boesze-Battaglia K. (2017). Phagocytosis-dependent ketogenesis in retinal pigment epithelium. J. Biol. Chem..

[53] Ma X., Wu W., Hara M., Zhou J., Panzarin C., Schafer C.M., Griffin C.T., Cai J., Ma J.-X., Takahashi Y. (2024). Deficient RPE mitochondrial energetics leads to subretinal fibrosis in age-related neovascular macular degeneration. Commun. Biol..

[54] Etchegaray J.I., Kelley S., Penberthy K., Karvelyte L., Nagasaka Y., Gasperino S., Paul S., Seshadri V., Raymond M., Royo Marco A. (2023). Phagocytosis in the retina promotes local insulin production in the eye. Nat. Metab..

[55] Etchegaray J.I., Ravichandran K. (2025). Role of RPE phagocytosis in the retina metabolic ecosystem. Adv. Exp. Med. Biol..

[56] Hass D.T., Giering E., Han J.Y.S., Bisbach C.M., Pandey K., Robbings B.M., Mundinger T., Nolan N., Tsang S., Peachey N. (2025). In vivo exchange of glucose and lactate between photoreceptors and the retinal pigment epithelium. eLife.

[57] Kanow M.A., Giarmarco M.M., Jankowski C.S.R., Tsantilas K., Engel A.L., Du J., Linton J.D., Farnsworth C.C., Sloat S.R., Rountree A. (2017). Biochemical adaptations of the retina and retinal pigment epithelium support a metabolic ecosystem in the vertebrate eye. eLife.

[58] Hurley J.B. (2021). Retina metabolism and metabolism in the pigmented epithelium: A busy intersection. Annu. Rev. Vis. Sci..

[59] Pan W.W., Wubben T.J., Besirli C.G. (2021). Photoreceptor metabolic reprogramming: Current understanding and therapeutic implications. Commun. Biol..

[60] Daniele L.L., Han J.Y.S., Samuels I.S., Komirisetty R., Mehta N., McCord J.L., Yu M., Wang Y., Boesze-Battaglia K., Bell B.A. (2022). Glucose uptake by GLUT1 in photoreceptors is essential for outer segment renewal and rod photoreceptor survival. FASEB J..

[61] Rajala R.V.S. (2020). Aerobic glycolysis in the retina: Functional roles of pyruvate kinase isoforms. Front. Cell Dev. Biol..

[62] Rajala A., Wang Y., Brush R.S., Tsantilas K., Jankowski C.S.R., Lindsay K.J., Linton J.D., Hurley J.B., Anderson R.E., Rajala R.V.S. (2018). Pyruvate kinase M2 regulates photoreceptor structure, function, and viability. Cell Death Dis..

[63] Zhang R., Shen W., Du J., Gillies M.C. (2020). Selective knockdown of hexokinase 2 in rods leads to age-related photoreceptor degeneration and retinal metabolic remodeling. Cell Death Dis..

[64] Weh E., Goswami M., Chaudhury S., Fernando R., Miller N., Hager H., Sheskey S., Sharma V., Wubben T.J., Besirli C.G. (2023). Metabolic alterations caused by simultaneous loss of HK2 and PKM2 leads to photoreceptor dysfunction and degeneration. Cells.

[65] Hanna J., David L.A., Touahri Y., Fleming T., Screaton R.A., Schuurmans C. (2022). Beyond genetics: The role of metabolism in photoreceptor survival, development and repair. Front. Cell Dev. Biol..

[66] Han J.Y.S., Kinoshita J., Bisetto S., Bell B.A., Nowak R.A., Peachey N.S., Philp N.J. (2020). Role of monocarboxylate transporters in regulating metabolic homeostasis in the outer retina: Insight gained from cell-specific Bsg deletion. FASEB J..

[67] Cozza G., Rossetto M., Bosello-Travain V., Maiorino M., Roveri A., Toppo S., Zaccarin M., Zennaro L., Ursini F. (2017). Glutathione peroxidase 4-catalyzed reduction of lipid hydroperoxides in membranes: The polar head of membrane phospholipids binds the enzyme and addresses the fatty acid hydroperoxide group toward the redox center. Free Radic. Biol. Med..

[68] Yang W.S., SriRamaratnam R., Welsch M.E., Shimada K., Skouta R., Viswanathan V.S., Cheah J.H., Clemons P.A., Shamji A.F., Clish C.B. (2014). Regulation of ferroptotic cancer cell death by GPX4. Cell.

[69] Knight L.J., Martis R.M., Donaldson P.J., Acosta M.L., Lim J.C. (2025). Changes in redox balance and mitochondrial activity in the retinas of cystine/glutamate antiporter knockout mice. Investig. Ophthalmol. Vis. Sci..

[70] Chen C., Wang H., Yang J., Zhao B., Lei Y., Li H., Yang K., Liu B., Diao Y. (2025). Sodium Iodate-Induced Ferroptosis in Photoreceptor-Derived 661W Cells Through the Depletion of GSH. Int. J. Mol. Sci..

[71] Sayyad Z., Sirohi K., Radha V., Swarup G. (2017). 661W is a retinal ganglion precursor-like cell line in which glaucoma-associated optineurin mutants induce cell death selectively. Sci. Rep..

[72] Brunet A.A., James R.E., Swanson P., Carvalho L.S. (2025). A review of the 661W cell line as a tool to facilitate treatment development for retinal diseases. Cell Biosci..

[73] Guo D., Sun Y., Wu J., Ding L., Jiang Y., Xue Y., Ma Y., Sun F. (2024). Photoreceptor-targeted extracellular vesicles-mediated delivery of Cul7 siRNA for retinal degeneration therapy. Theranostics.

[74] Tang W., Zhai R., Ma J., Xu G. (2025). Lipocalin-2-mediated ferroptosis as a target for protection against light-induced photoreceptor degeneration. Mol. Med..

[75] Zeng L., Hu P., Wang X., Ding X., Wang Q., Luo L., Zhang Y., Li M., Zhao Y., Li S. (2025). Sirtuin-3 activation by honokiol attenuated anesthesia/surgery-induced cognitive impairment and neuronal ferroptosis via inhibiting mitochondrial GPX4 acetylation. J. Nanobiotechnol..

[76] Ban N., Ozawa Y., Osada H., Lin J.B., Toda E., Watanabe M., Yuki K., Kubota S., Apte R.S., Tsubota K. (2017). Neuroprotective role of retinal SIRT3 against acute photo-stress. npj Aging Mech. Dis..

[77] Cao J., Chen X., Chen L., Lu Y., Wu Y., Deng A., Pan F., Huang H., Liu Y., Li Y. (2025). DHODH-mediated mitochondrial redox homeostasis: A novel ferroptosis regulator and promising therapeutic target. Redox Biol..

[78] Orozco Rodriguez J.M., Wacklin-Knecht H.P., Clifton L.A., Bogojevic O., Leung A., Fragneto G., Knecht W. (2022). New insights into the interaction of class II dihydroorotate dehydrogenases with ubiquinone in lipid bilayers as a function of lipid composition. Int. J. Mol. Sci..

[79] Gan B. (2021). Mitochondrial regulation of ferroptosis. J. Cell Biol..

[80] Deng R., Fu L., Liang H., Ai X., Liu F., Li N., Wu L., Li S., Yang X., Lin Y. (2025). Inhibition of mitochondrial complex I induces mitochondrial ferroptosis by regulating CoQH_2_ levels in cancer. Cell Death Dis..

[81] Wu M.-F., Peng X., Zhang M.-C., Guo H., Xie H.-T. (2025). Ferroptosis and PANoptosis under hypoxia pivoting on the crosstalk between DHODH and GPX4 in corneal epithelium. Free Radic. Biol. Med..

[82] Doll S., Freitas F.P., Shah R., Aldrovandi M., da Silva M.C., Ingold I., Grocin A.G., Xavier da Silva T.N., Panzilius E., Scheel C.H. (2019). FSP1 is a glutathione-independent ferroptosis suppressor. Nature.

[83] Bersuker K., Hendricks J.M., Li Z., Magtanong L., Ford B., Tang P.H., Roberts M.A., Tong B., Maimone T.J., Zoncu R. (2019). The CoQ oxidoreductase FSP1 acts parallel to GPX4 to inhibit ferroptosis. Nature.

[84] Li X., Zhu S., Qi F. (2023). Blue light pollution causes retinal damage and degeneration by inducing ferroptosis. J. Photochem. Photobiol. B.

[85] Dai X., Yang X., Feng Y., Wu X., Ju Y., Zou R., Yuan F. (2025). The role of vitamin K and its antagonist in the process of ferroptosis-damaged RPE-mediated CNV. Cell Death Dis..

[86] Méjécase C., Zhou Y., Owen N., Soro-Barrio P., Cheloni R., Nair N., Sarkar H., Toualbi L., Moosajee M. (2025). Dominant RDH12-retinitis pigmentosa impairs photoreceptor development and implicates cone involvement in retinal organoids. Front. Cell Dev. Biol..

[87] Kraft V.A.N., Bezjian C.T., Pfeiffer S., Ringelstetter L., Müller C., Zandkarimi F., Merl-Pham J., Bao X., Anastasov N., Kössl J. (2020). GTP Cyclohydrolase 1/Tetrahydrobiopterin Counteract Ferroptosis through Lipid Remodeling. ACS Cent. Sci..

[88] Edgar K.S., Cunning C., Gardiner T.A., McDonald D.M. (2023). BH4 supplementation reduces retinal cell death in ischaemic retinopathy. Sci. Rep..

[89] Landowski M., Hagimori R., Gogoi P., Shahi P.K., Oikawa K., Bhute V.J., McLellan G.J., Ikeda S., Yamada K., Pattnaik B.R. (2026). Mitofusins are required for specialized mitochondrial morphology and function of rod photoreceptor cells. Front. Cell Dev. Biol..

[90] Nyenhuis S.B., Wu X., Strub M.-P., Yim Y.-I., Stanton A.E., Baena V., Syed Z.A., Canagarajah B., Hammer J.A., Hinshaw J.E. (2023). OPA1 helical structures give perspective to mitochondrial dysfunction. Nature.

[91] Han S., Zhao F., Hsia J., Ma X., Liu Y., Torres S., Fujioka H., Zhu X. (2021). The role of Mfn2 in the structure and function of endoplasmic reticulum–mitochondrial tethering in vivo. J. Cell Sci..

[92] Zhang Z., Zhou H., Gu W., Wei Y., Mou S., Wang Y., Zhang J., Zhong Q. (2024). CGI1746 targets σ1R to modulate ferroptosis through mitochondria-associated membranes. Nat. Chem. Biol..

[93] Xu L., Xu Y., Jiang Y., Jiang J., Chen S., Sun D., Li S., Wei F., Zhu H. (2024). IP3R2 regulates apoptosis by Ca^2+^ transfer through mitochondria–ER contacts in hypoxic photoreceptor injury. Exp. Eye Res..

[94] Campos J., Gleitze S., Hidalgo C., Núñez M.T. (2024). IP3R-mediated calcium release promotes ferroptotic death in SH-SY5Y neuroblastoma cells. Antioxidants.

[95] Pedrera L., Prieto Clemente L., Dahlhaus A., Lotfipour Nasudivar S., Tishina S., Olmo González D., Stroh J., Yapici F.I., Singh R.P., Grotehans N. (2025). Ferroptosis triggers mitochondrial fragmentation via Drp1 activation. Cell Death Dis..

[96] Tang S., Fuß A., Fattahi Z., Culmsee C. (2024). Drp1 depletion protects against ferroptotic cell death by preserving mitochondrial integrity and redox homeostasis. Cell Death Dis..

[97] Ma S., Qin J., Zhang Y., Luan J., Sun N., Hou G., He J., Xiao Y., Zhang W., Gao M. (2025). Disrupting mitochondrial dynamics attenuates ferroptosis and chemotoxicity via upregulating NRF2-mediated FSP1 expression. Cell Rep..

[98] Tang S., Huang M., Wang R., Li M., Dong N., Wu R., Chi Z., Gao L. (2024). Drp1-dependent mitochondrial fragmentation mediates photoreceptor abnormalities in type 1 diabetic retina. Exp. Eye Res..

[99] Fisher C.R., Shaaeli A.A., Ebeling M.C., Montezuma S.R., Ferrington D.A. (2022). Investigating mitochondrial fission, fusion, and autophagy in retinal pigment epithelium from donors with age-related macular degeneration. Sci. Rep..

[100] Qiu S., Zhong X., Meng X., Li S., Qian X., Lu H., Cai J., Zhang Y., Wang M., Ye Z. (2023). Mitochondria-localized cGAS suppresses ferroptosis to promote cancer progression. Cell Res..

[101] Zou M., Ke Q., Nie Q., Qi R., Zhu X., Liu W., Hu X., Sun Q., Fu J.L., Tang X. (2022). Inhibition of cGAS-STING by JQ1 alleviates oxidative stress-induced retina inflammation and degeneration. Cell Death Differ..

[102] Li D., Chang J., Wang Y., Du X., Xu J., Cui J., Zhang T., Chen Y. (2024). Hyperoside mitigates photoreceptor degeneration in part by targeting cGAS and suppressing DNA-induced microglial activation. Acta Neuropathol. Commun..

[103] Li J., Yang D., Li Z., Zhao M., Wang D., Sun Z., Wen P., Dai Y., Gou F., Ji Y. (2023). PINK1/Parkin-mediated mitophagy in neurodegenerative diseases. Ageing Res. Rev..

[104] Okatsu K., Fukai S. (2026). Ubiquitin signaling in PINK1/Parkin-dependent mitophagy. J. Biochem..

[105] Zapata-Muñoz J., Jiménez-Loygorri J.I., Stumpe M., Villarejo-Zori B., Alonso-Gil S., Terešak P., Mathai B.J., Ganley I.G., Simonsen A., Dengjel J. (2025). The developing retina undergoes mitochondrial remodeling via PINK1/PRKN-dependent mitophagy. J. Mol. Biol..

[106] Zhou B., Fang L., Dong Y., Yang J., Chen X., Zhang N., Zhu Y., Huang T. (2021). Mitochondrial quality control protects photoreceptors against oxidative stress in the H_2_O_2_-induced models of retinal degeneration diseases. Cell Death Dis..

[107] Sridevi Gurubaran I., Viiri J., Koskela A., Hyttinen J.M.T., Paterno J.J., Kis G., Antal M., Urtti A., Kauppinen A., Felszeghy S. (2020). Mitophagy in the retinal pigment epithelium of dry age-related macular degeneration investigated in the NFE2L2/PGC-1α−/− mouse model. Int. J. Mol. Sci..

[108] Feng X., Cai W., Li Q., Zhao L., Meng Y., Xu H. (2025). Activation of lysosomal Ca^2+^ channels mitigates mitochondrial damage and oxidative stress. J. Cell Biol..

[109] Tan L.X., Germer C.J., Thamban T., La Cunza N., Lakkaraju A. (2023). Optineurin tunes outside-in signaling to regulate lysosome biogenesis and phagocytic clearance in the retina. Curr. Biol..

[110] Abu Shelbayeh O., Arroum T., Morris S., Busch K.B. (2023). PGC-1α Is a Master Regulator of Mitochondrial Lifecycle and ROS Stress Response. Antioxidants.

[111] Hu C., Ren C., Wu Y., Lin R., Shen T., Li T., Yu D., Jiang L., Wan Z., Luo Y. (2025). ZLN005, a PGC-1α agonist, delays photoreceptor degeneration by enhancing mitochondrial biogenesis in a murine model of retinitis pigmentosa. Neuropharmacology.

[112] Ozawa Y., Toda E., Homma K., Osada H., Nagai N., Tsubota K., Okano H. (2022). Effects of Epigenetic Modification of PGC-1α by a Chemical Chaperon on Mitochondria Biogenesis and Visual Function in Retinitis Pigmentosa. Cells.

[113] Kiyama T., Chen C.-K., Wang S.W., Pan P., Ju Z., Wang J., Takada S., Klein W.H., Mao C.-A. (2018). Essential roles of mitochondrial biogenesis regulator Nrf1 in retinal development and homeostasis. Mol. Neurodegener..

[114] Zhou S., Taskintuna K., Hum J., Gulati J., Olaya S., Steinman J., Golestaneh N. (2024). PGC-1α repression dysregulates lipid metabolism and induces lipid droplet accumulation in the retinal pigment epithelium. Cell Death Dis..

[115] Dhivya M.A., Aberami S., Nikhalashree S., Biswas J., Liu W., Irudayaraj J., Sulochana K.N., Coral K., Bharathi Devi S.R. (2020). Copper mediates mitochondrial biogenesis in retinal pigment epithelial cells. Biochim. Biophys. Acta Mol. Basis Dis..

[116] Shi R., Hou W., Wang Z.Q., Xu X. (2021). Biogenesis of iron–sulfur clusters and their role in DNA metabolism. Front. Cell Dev. Biol..

[117] Zhang W., Xu L., Zhao H., Li K. (2021). Mammalian mitochondrial iron–sulfur cluster biogenesis and transfer and related human diseases. Biophys. Rep..

[118] Shahandeh A., Bui B.V., Finkelstein D.I., Nguyen C.T.O. (2022). Effects of Excess Iron on the Retina: Insights from Clinical Cases and Animal Models of Iron Disorders. Front. Neurosci..

[119] Mancias J.D., Wang X., Gygi S.P., Harper J.W., Kimmelman A.C. (2014). Quantitative proteomics identifies NCOA4 as the cargo receptor mediating ferritinophagy. Nature.

[120] Gao M., Monian P., Pan Q., Zhang W., Xiang J., Jiang X. (2016). Ferroptosis is an autophagic cell death process. Cell Res..

[121] Mi D., Yanatori I., Zheng H., Kong Y., Hirayama T., Toyokuni S. (2024). Association of poly(rC)-binding protein-2 with sideroflexin-3 through TOM20 as an iron entry pathway to mitochondria. Free Radic. Res..

[122] Chen B., Aredo B., Ding Y., Zhong X., Zhu Y., Zhao C.X., Kumar A., Xing C., Gautron L., Lyon S. (2020). Forward genetic analysis using OCT screening identifies Sfxn3 mutations leading to progressive outer retinal degeneration in mice. Proc. Natl. Acad. Sci. USA.

[123] Hryc C.F., Mallampalli V.K.P.S., Bovshik E.I., Azinas S., Fan G., Serysheva I.I., Sparagna G.C., Baker M.L., Mileykovskaya E., Dowhan W. (2023). Structural insights into cardiolipin replacement by phosphatidylglycerol in a cardiolipin-lacking yeast respiratory supercomplex. Nat. Commun..

[124] Jadhav S., Protchenko O., Li F., Baratz E., Shakoury-Elizeh M., Maschek A., Cox J., Philpott C.C. (2021). Mitochondrial dysfunction in mouse livers depleted of iron chaperone PCBP1. Free Radic. Biol. Med..

[125] Totsuka K., Ueta T., Uchida T., Roggia M.F., Nakagawa S., Vavvas D.G., Honjo M., Aihara M. (2019). Oxidative Stress Induces Ferroptotic Cell Death in Retinal Pigment Epithelial Cells. Exp. Eye Res..

[126] Fedotcheva T., Shimanovsky N., Fedotcheva N. (2023). Specific Features of Mitochondrial Dysfunction under Conditions of Ferroptosis Induced by t-Butylhydroperoxide and Iron: Protective Role of the Inhibitors of Lipid Peroxidation and Mitochondrial Permeability Transition Pore Opening. Membranes.

[127] Hou T., Fan X., Zhang Q., Zhang H., Zhang D., Tao L., Wang Z. (2024). Dibutyl phthalate exposure induced mitochondria-dependent ferroptosis by enhancing VDAC2 in zebrafish ZF4 cells. Environ. Pollut..

[128] Ye T., Yang W., Gao T., Yu X., Chen T., Yang Y., Guo J., Li Q., Li H., Yang L. (2023). Trastuzumab-induced cardiomyopathy via ferroptosis-mediated mitochondrial dysfunction. Free Radic. Biol. Med..

[129] Yang J., Lu X., Hao J.-L., Li L., Ruan Y.-T., An X.-N., Huang Q.-L., Dong X.-M., Gao P. (2025). VSTM2L protects prostate cancer cells against ferroptosis via inhibiting VDAC1 oligomerization and maintaining mitochondria homeostasis. Nat. Commun..

[130] Jang S.K., Ahn S.H., Kim G., Kim S., Hong J., Park K.S., Park I.C., Jin H.O. (2024). Inhibition of VDAC1 oligomerization blocks cysteine deprivation-induced ferroptosis via mitochondrial ROS suppression. Cell Death Dis..

[131] Wan H., Ban X., He Y., Yang Y., Hu X., Shang L., Wan X., Zhang Q., Xiong K. (2026). Voltage-dependent anion channel 1 oligomerization regulates PANoptosis in retinal ischemia–reperfusion injury. Neural Regen. Res..

[132] Karlstetter M., Nothdurfter C., Aslanidis A., Moeller K., Horn F., Scholz R., Neumann H., Weber B.H.F., Rupprecht R., Langmann T. (2014). Translocator protein (18 kDa) (TSPO) is expressed in reactive retinal microglia and modulates microglial inflammation and phagocytosis. J. Neuroinflammation.

[133] Wolf A., Herb M., Schramm M., Langmann T. (2020). The TSPO-NOX1 axis controls phagocyte-triggered pathological angiogenesis in the eye. Nat. Commun..

[134] Corsi F., Castagnoli J., Galante A., Fabiano A., Nuti E., Piras A.M., Taliani S., Piano I., Gargini C. (2025). TSPO modulation prevents photoreceptor degeneration and produces neuroprotective effects in an animal model of retinitis pigmentosa. Cells.

[135] Corsi F., Baglini E., Barresi E., Salerno S., Cerri C., Martini C., Da Settimo F., Taliani S., Gargini C., Piano I. (2022). Targeting TSPO reduces inflammation and apoptosis in an in vitro photoreceptor-like model of retinal degeneration. ACS Chem. Neurosci..

[136] Lejri I., Grimm A., Hallé F., Abarghaz M., Klein C., Maitre M., Schmitt M., Bourguignon J.J., Mensah-Nyagan A.G., Bihel F. (2019). TSPO Ligands Boost Mitochondrial Function and Pregnenolone Synthesis. J. Alzheimer’s Dis..

[137] Patel P., Mendoza A., Robichaux D.J., Wang M.C., Wehrens X.H.T., Karch J. (2021). Inhibition of the anti-apoptotic Bcl-2 family by BH3 mimetics sensitize the mitochondrial permeability transition pore through Bax and Bak. Front. Cell Dev. Biol..

[138] Qiu Y., Hüther J.A., Wank B., Rath A., Tykwe R., Aldrovandi M., Henkelmann B., Mergner J., Nakamura T., Laschat S. (2025). Interplay of ferroptotic and apoptotic cell death and its modulation by BH3-mimetics. Cell Death Differ..

[139] Ma J.Y.W., Greferath U., Wong J.H.C., Fothergill L.J., Jobling A.I., Vessey K.A., Fletcher E.L. (2023). Aging induces cell loss and a decline in phagosome processing in the mouse retinal pigment epithelium. Neurobiol. Aging.

[140] Sayama A., Okado K., Nakamura K., Kawaguchi T., Iguchi T., Makino T., Yabe K., Kai K., Mori K. (2018). UNC569-induced morphological changes in pigment epithelia and photoreceptor cells in the retina through MerTK inhibition in mice. Toxicol. Pathol..

[141] Lew D.S., Mazzoni F., Finnemann S.C. (2020). Microglia inhibition delays retinal degeneration due to MerTK phagocytosis receptor deficiency. Front. Immunol..

[142] Krohne T.U., Stratmann N.K., Kopitz J., Holz F.G. (2010). Effects of lipid peroxidation products on lipofuscinogenesis and autophagy in human retinal pigment epithelial cells. Exp. Eye Res..

[143] Escrevente C., Falcão A.S., Hall M.J., Lopes-da-Silva M., Antas P., Mesquita M.M., Ferreira I.S., Cardoso M.H., Oliveira D., Fradinho A.C. (2021). Formation of lipofuscin-like autofluorescent granules in the retinal pigment epithelium requires lysosome dysfunction. Investig. Ophthalmol. Vis. Sci..

[144] Pan C., Banerjee K., Lehmann G., Almeida D., Hajjar K.A., Benedicto I., Jiang Z., Radu R.A., Thompson D.H., Rodriguez-Boulan E. (2021). Lipofuscin causes atypical necroptosis through lysosomal membrane permeabilization. Proc. Natl. Acad. Sci. USA.

[145] Ashok A., Chaudhary S., Wise A.S., Rana N.A., McDonald D., Kritikos A.E., Lindner E., Singh N. (2021). Release of Iron-Loaded Ferritin in Sodium Iodate-Induced Model of Age Related Macular Degeneration: An In-Vitro and In-Vivo Study. Antioxidants.

[146] Chen M., Wang Y., Dalal R., Du J., Vollrath D. (2024). Alternative oxidase blunts pseudohypoxia and photoreceptor degeneration due to RPE mitochondrial dysfunction. Proc. Natl. Acad. Sci. USA.

[147] Dobreva A., Camacho E.T., Miranda M. (2023). Mathematical model for glutathione dynamics in the retina. Sci. Rep..

[148] Kim H.J., Sparrow J.R. (2021). Bisretinoid phospholipid and vitamin A aldehyde: Shining a light. J. Lipid Res..

[149] Farnoodian M., Bose D., Barone F., Nelson L.M., Boyle M., Jun B., Do K., Gordon W., Kautzmann Guerin M.-A., Perera R. (2023). Retina and RPE lipid profile changes linked with ABCA4 associated Stargardt’s maculopathy. Pharmacol. Ther..

[150] Chen C., Yang K., He D., Yang B., Tao L., Chen J., Wu Y. (2023). Induction of ferroptosis by HO-1 contributes to retinal degeneration in mice with defective clearance of all-trans-retinal. Free Radic. Biol. Med..

[151] Yang B., Yang K., Xi R., Li S., Chen J., Wu Y. (2025). Inhibition of JNK signaling attenuates photoreceptor ferroptosis caused by all-trans-retinal. Free Radic. Biol. Med..

[152] Yang B., Yang K., Chen Y., Li Q., Chen J., Li S., Wu Y. (2025). Exposure of A2E to blue light promotes ferroptosis in the retinal pigment epithelium. Cell. Mol. Biol. Lett..

[153] Azuma K., Suzuki T., Kobayashi K., Nagahara M., Imai H., Suga A., Iwata T., Shiraya T., Aihara M., Ueta T. (2024). Retinal pigment epithelium-specific ablation of GPx4 in adult mice recapitulates key features of geographic atrophy in age-related macular degeneration. Cell Death Dis..

[154] Tang W., Guo J., Liu W., Ma J., Xu G. (2021). Ferrostatin-1 attenuates ferroptosis and protects the retina against light-induced retinal degeneration. Biochem. Biophys. Res. Commun..

[155] Yang B., Yang K., Chen Y., Xi R., Han J., Li S., Chen J., Wu Y. (2025). Activation of GSDME by all-trans-retinal increases sensitivity to photoreceptor ferroptosis. Int. J. Biol. Sci..

[156] Song D., Song Y., Hadziahmetovic M., Zhong Y., Dunaief J.L. (2012). Systemic administration of the iron chelator deferiprone protects against light-induced photoreceptor degeneration in the mouse retina. Free Radic. Biol. Med..

[157] Obolensky A., Berenshtein E., Lederman M., Bulvik B., Alper-Pinus R., Yaul R., Deleon E., Chowers I., Chevion M., Banin E. (2011). Zinc–desferrioxamine attenuates retinal degeneration in the rd10 mouse model of retinitis pigmentosa. Free Radic. Biol. Med..

[158] Lu L., Oveson B.C., Jo Y.J., Lauer T.W., Usui S., Komeima K., Xie B., Campochiaro P.A. (2009). Increased expression of glutathione peroxidase 4 strongly protects retina from oxidative damage. Antioxid. Redox Signal..

[159] Zheng J., Zhang W., Ito J., Henkelmann B., Xu C., Mishima E., Conrad M. (2025). N-acetyl-L-cysteine averts ferroptosis by fostering glutathione peroxidase 4. Cell Chem. Biol..

[160] Campochiaro P.A., Iftikhar M., Hafiz G., Akhlaq A., Tsai G., Wehling D., Lu L., Wall G.M., Singh M.S., Kong X. (2020). Oral N-acetylcysteine improves cone function in retinitis pigmentosa patients in phase I trial. J. Clin. Investig..

[161] Liu Y., Bell B.A., Song Y., Zhang K., Anderson B., Axelsen P.H., Bohannan W., Agbaga M.-P., Park H.G., James G. (2022). Deuterated docosahexaenoic acid protects against oxidative stress and geographic atrophy-like retinal degeneration in a mouse model with iron overload. Aging Cell.

[162] Chew E.Y., Clemons T.E., Agrón E., Domalpally A., Keenan T.D.L., Vitale S., Weber C., Smith D.C., Christianson D.J., Bressler S.B. (2022). Long-term outcomes of adding lutein/zeaxanthin and ω-3 fatty acids to the AREDS supplements on age-related macular degeneration progression: AREDS2 Report 28. JAMA Ophthalmol..

[163] Lunegova D.A., Gvozdev D.A., Senin I.I., Gudkova V.R., Sidorenko S.V., Tiulina V.V., Shebardina N.G., Yakovleva M.A., Feldman T.B., Ramonova A.A. (2025). Antioxidant properties of the soluble carotenoprotein AstaP and its feasibility for retinal protection against oxidative stress. FEBS J..

[164] Yang Y., Wang Y., Deng Y., Lu J., Xiao L., Li J., Zhou Y., Nie F., Chen X., Peng J. (2023). *Fructus Lycii* and *Salvia miltiorrhiza* Bunge extract attenuate oxidative stress-induced photoreceptor ferroptosis in retinitis pigmentosa. Biomed. Pharmacother..

[165] Zhao Y., Li Q., Jian W., Han X., Zhang Y., Zeng Y., Liu R., Wang Q., Song Q. (2023). Protective benefits of salvianic acid A against retinal iron overload by inhibition of ferroptosis. Biomed. Pharmacother..

[166] Malik N., Shaw R.J. (2025). The AMPK Pathway: Molecular Rejuvenation of Metabolism and Mitochondria. Annu. Rev. Cell Dev. Biol..

[167] Xu L., Kong L., Wang J., Ash J.D. (2018). Stimulation of AMPK prevents degeneration of photoreceptors and the retinal pigment epithelium. Proc. Natl. Acad. Sci. USA.

[168] Song S., Bao S., Zhang C., Zhang J., Lv J., Li X., Chudhary M., Ren X., Kong L. (2021). Stimulation of AMPK prevents diabetes-induced photoreceptor cell degeneration. Oxidative Med. Cell. Longev..

[169] Kawashima H., Ozawa Y., Toda E., Homma K., Osada H., Narimatsu T., Nagai N., Tsubota K. (2020). Neuroprotective and vision-protective effect of preserving ATP levels by AMPK activator. FASEB J..

[170] Zhang X., Henneman N.F., Girardot P.E., Sellers J.T., Chrenek M.A., Li Y., Wang J., Brenner C., Nickerson J.M., Boatright J.H. (2020). Systemic treatment with nicotinamide riboside is protective in a mouse model of light-induced retinal degeneration. Investig. Ophthalmol. Vis. Sci..

[171] Chen X., Amorim J.A., Moustafa G.A., Lee J.-J., Yu Z., Ishihara K., Iesato Y., Barbisan P., Ueta T., Togka K.A. (2020). Neuroprotective effects and mechanisms of action of nicotinamide mononucleotide in a photoreceptor degenerative model of retinal detachment. Aging.

[172] Yan Y., Wang Y., Ding J., Lu L., Ke G.J., Dong K. (2021). TRPML1 inhibited photoreceptor apoptosis and protected the retina by activation of autophagy in experimental retinal detachment. Ophthalmic Res..

[173] Rosdah A.A., Abbott B.M., Langendorf C.G., Deng Y., Truong J.Q., Waddell H.M.M., Ling N.X.Y., Smiles W.J., Delbridge L.M.D., Liu G.S. (2022). A novel small molecule inhibitor of human Drp1. Sci. Rep..

[174] Bordt E.A., Clerc P., Roelofs B.A., Saladino A.J., Tretter L., Adam-Vizi V., Cherok E., Khalil A., Yadava N., Ge S.X. (2017). The putative Drp1 inhibitor mdivi-1 is a reversible mitochondrial complex I inhibitor that modulates reactive oxygen species. Dev. Cell.

[175] Marx N., Ritter N., Disse P., Seebohm G., Busch K.B. (2024). Detailed analysis of Mdivi-1 effects on complex I and respiratory supercomplex assembly. Sci. Rep..

[176] Carrella S., Di Guida M., Brillante S., Piccolo D., Ciampi L., Guadagnino I., Garcia Piqueras J., Pizzo M., Marrocco E., Molinari M. (2022). miR-181a/b downregulation: A mutation-independent therapeutic approach for inherited retinal diseases. EMBO Mol. Med..

[177] She X., Lu X., Li T., Sun J., Liang J., Zhai Y., Yang S., Gu Q., Wei F., Zhu H. (2018). Inhibition of mitochondrial fission preserves photoreceptors after retinal detachment. Am. J. Pathol..

[178] Liu Z., Ma J., Zuo X., Zhang X., Xie H., Wang F., Wu C., Zhang J., Zhu Q. (2023). IP3R-dependent mitochondrial dysfunction mediates C5b-9-induced ferroptosis in trichloroethylene-caused immune kidney injury. Front. Immunol..

[179] Ehlers J.P., Hu A., Boyer D., Cousins S.W., Waheed N.K., Rosenfeld P.J., Brown D., Kaiser P.K., Abbruscato A., Gao G. (2025). ReCLAIM-2: A randomized phase II clinical trial evaluating elamipretide in age-related macular degeneration, geographic atrophy growth, visual function, and ellipsoid zone preservation. Ophthalmol. Sci..

[180] Adijanto J., Du J., Moffat C., Seifert E.L., Hurley J.B., Philp N.J. (2014). The retinal pigment epithelium utilizes fatty acids for ketogenesis: Implications for metabolic coupling with the outer retina. J. Biol. Chem..

[181] Yako T., Nakamura M., Otsu W., Nakamura S., Shimazawa M., Hara H. (2021). Mitochondria dynamics in the aged mice eye and the role in the RPE phagocytosis. Exp. Eye Res..

[182] Deng W.T., Dinculescu A., Li Q., Boye S.L., Li J., Gorbatyuk M.S., Pang J., Chiodo V., Liu L., Alkuraya F. (2012). Tyrosine-mutant AAV8 delivery of human MERTK provides long-term retinal preservation in RCS rats. Investig. Ophthalmol. Vis. Sci..

[183] Testa F., Bacci G., Falsini B., Iarossi G., Melillo P., Mucciolo D., Murro V., Salvetti A., Sodi A., Staurenghi G. (2024). Voretigene neparvovec for inherited retinal dystrophy due to RPE65 mutations: A scoping review of eligibility and treatment challenges from clinical trials to real practice. Eye.

[184] Maguire A.M., Russell S., Chung D.C., Yu Z.F., Tillman A., Drack A.V., Simonelli F., Leroy B.P., Reape K.Z., High K.A. (2021). Durability of voretigene neparvovec for biallelic RPE65-mediated inherited retinal disease: Phase 3 results at 3 and 4 years. Ophthalmology.

[185] Liu Y., Bell B.A., Song Y., Kim H.J., Sterling J.K., Kim B.J., Poli M., Guo M., Zhang K., Rao A. (2021). Intraocular iron injection induces oxidative stress followed by elements of geographic atrophy and sympathetic ophthalmia. Aging Cell.

[186] Youale J., Bigot K., Jaworski T., Lebon C., Françon A., Delaunay K., Bénard R., De Bastard T., Daruich A., Kaddour N. (2025). Transferrin is a drug candidate for the treatment of dry age-related macular degeneration (AMD). Cell Death Dis..

[187] Bigot K., Gondouin P., Bénard R., Montagne P., Youale J., Piazza M., Picard E., Bordet T., Behar-Cohen F. (2020). Transferrin non-viral gene therapy for treatment of retinal degeneration. Pharmaceutics.

[188] Kauper K., Nystuen A., Orecchio L., Gonzalez-Lopez E., Lee A., Duncan J.L., Stewart J.M., Aaberg T. (2025). Long-Term Durability of Ciliary Neurotrophic Factor-Releasing Revakinagene Taroretcel-lwey in Individuals with Retinal Degenerative Disorders. Investig. Ophthalmol. Vis. Sci..

[189] Chew E.Y., Gillies M., Jaffe G.J., Gaudric A., Egan C., Constable I., Clemons T., Aaberg T., Manning D.C., Hohman T.C. (2025). Cell-based ciliary neurotrophic factor therapy for macular telangiectasia type 2. NEJM Evid..

[190] Hansman D.S., Du J., Casson R.J., Peet D.J. (2025). Eye on the horizon: The metabolic landscape of the RPE in aging and disease. Prog. Retin. Eye Res..

[191] Zekavat S.M., Sekimitsu S., Ye Y., Raghu V.K., Zhao H., Elze T., Segrè A.V., Wiggs J.L., Natarajan P., Del Priore L.V. (2022). Photoreceptor Layer Thinning Is an Early Biomarker for Age-Related Macular Degeneration: Epidemiologic and Genetic Evidence from UK Biobank OCT Data. Ophthalmology.

[192] Le D., Son T., Lim J.I., Yao X. (2022). Quantitative Optical Coherence Tomography Reveals Rod Photoreceptor Degeneration in Early Diabetic Retinopathy. Retina.

[193] Cvekl A., Vijg J. (2024). Aging of the eye: Lessons from cataracts and age-related macular degeneration. Ageing Res. Rev..

[194] Bighinati A., Adani E., Stanzani A., D’Alessandro S., Marigo V. (2024). Molecular mechanisms underlying inherited photoreceptor degeneration as targets for therapeutic intervention. Front. Cell. Neurosci..

[195] Karademir D., Todorova V., Ebner L.J.A., Samardzija M., Grimm C. (2022). Single-cell RNA sequencing of the retina in a model of retinitis pigmentosa reveals early responses to degeneration in rods and cones. BMC Biol..

[196] Ye Z., Yan Y., Jin F., Jiang J., Deng C., Wang L., Dong K. (2025). Deferiprone protects photoreceptors by inhibiting ferroptosis after experimental retinal detachment. Exp. Eye Res..

[197] Song Q., Jian W., Zhang Y., Li Q., Zhao Y., Liu R., Zeng Y., Zhang F., Duan J. (2024). Puerarin Attenuates Iron Overload-Induced Ferroptosis in Retina through a Nrf2-Mediated Mechanism. Mol. Nutr. Food Res..

[198] Li Q., Zhang Y., Liu P., Wang C., Pan Y., Nie Y., Tang W., Wang Q., Song Q. (2024). Astragaloside IV attenuates ferroptosis and protects against iron overload-induced retinal injury. Exp. Eye Res..

[199] Kong X., Hafiz G., Wehling D., Akhlaq A., Campochiaro P.A. (2021). Locus-Level Changes in Macular Sensitivity in Patients with Retinitis Pigmentosa Treated with Oral N-Acetylcysteine. Am. J. Ophthalmol..

[200] Shen X., Chen Y., He B., Xi R., Chen J., Wu Y. (2025). Ferrostatin-1, a ferroptosis inhibitor, mitigates all-trans-retinal-induced retinal pigment epithelium degeneration in mice. J. Transl. Med..

